# The Bicocca sampling days model: a participatory citizen science approach to environmental microbiome research and education

**DOI:** 10.1093/ismeco/ycaf220

**Published:** 2025-11-22

**Authors:** Giulia Ghisleni, Alice Armanni, Sara Fumagalli, Asia Rosatelli, Yodit M Bacchi, Cristian Barillari, Denyse A Battista, Michela Benocci, Aurora Brunelli, Federica Cammarano, Giorgio Bovolini, Fabio Capuano, Noemi Bulla, Althea Colombo, Laura Colombo, Luca Corneo, Davide Evangelista, Piero Giorgetti, Giada Marin, Alessia Meziu, Mattia Riva, Davide Rizzo, Simone Romano, Stefano Ronchi, Riccardo Rossi, Roberta Volpi, Marta Zanotti, Marco Zenaro, Andrea Franzetti, Maurizio Casiraghi, Antonia Bruno

**Affiliations:** Department of Biotechnology and Biosciences, University of Milano-Bicocca, Milan 20126, Lombardy, Italy; Department of Biotechnology and Biosciences, University of Milano-Bicocca, Milan 20126, Lombardy, Italy; Department of Biotechnology and Biosciences, University of Milano-Bicocca, Milan 20126, Lombardy, Italy; Department of Earth and Environmental Sciences, University of Milano-Bicocca, Milan 20126, Lombardy, Italy; Department of Biotechnology and Biosciences, University of Milano-Bicocca, Milan 20126, Lombardy, Italy; Department of Biotechnology and Biosciences, University of Milano-Bicocca, Milan 20126, Lombardy, Italy; Department of Biotechnology and Biosciences, University of Milano-Bicocca, Milan 20126, Lombardy, Italy; Department of Earth and Environmental Sciences, University of Milano-Bicocca, Milan 20126, Lombardy, Italy; Department of Biotechnology and Biosciences, University of Milano-Bicocca, Milan 20126, Lombardy, Italy; Department of Biotechnology and Biosciences, University of Milano-Bicocca, Milan 20126, Lombardy, Italy; Department of Biotechnology and Biosciences, University of Milano-Bicocca, Milan 20126, Lombardy, Italy; Department of Biotechnology and Biosciences, University of Milano-Bicocca, Milan 20126, Lombardy, Italy; Department of Biotechnology and Biosciences, University of Milano-Bicocca, Milan 20126, Lombardy, Italy; Department of Biotechnology and Biosciences, University of Milano-Bicocca, Milan 20126, Lombardy, Italy; Department of Biotechnology and Biosciences, University of Milano-Bicocca, Milan 20126, Lombardy, Italy; Department of Biotechnology and Biosciences, University of Milano-Bicocca, Milan 20126, Lombardy, Italy; Department of Oncology and Hemato-oncology, University of Milan, Milan 20122, Lombardy, Italy; Department of Chemistry, Materials and Chemical Engineering “G.Natta”, Politecnico di Milano, Milan 20133, Lombardy, Italy; Fondazione Policlinico Universitario Agostino Gemelli IRCSS, Rome 00168, Lazio, Italy; Dipartment di Neuroscience, University Cattolica del Sacro Cuore, Rome 00168, Lazio, Italy; Department of Biotechnology and Biosciences, University of Milano-Bicocca, Milan 20126, Lombardy, Italy; Department of Biotechnology and Biosciences, University of Milano-Bicocca, Milan 20126, Lombardy, Italy; Department of Biotechnology and Biosciences, University of Milano-Bicocca, Milan 20126, Lombardy, Italy; Department of Biotechnology and Biosciences, University of Milano-Bicocca, Milan 20126, Lombardy, Italy; Department of Biotechnology and Biosciences, University of Milano-Bicocca, Milan 20126, Lombardy, Italy; Department of Biotechnology and Biosciences, University of Milano-Bicocca, Milan 20126, Lombardy, Italy; Department of Biotechnology and Biosciences, University of Milano-Bicocca, Milan 20126, Lombardy, Italy; Department of Biotechnology and Biosciences, University of Milano-Bicocca, Milan 20126, Lombardy, Italy; Department of Biotechnology and Biosciences, University of Milano-Bicocca, Milan 20126, Lombardy, Italy; Department of Biotechnology and Biosciences, University of Milano-Bicocca, Milan 20126, Lombardy, Italy; Department of Biotechnology and Biosciences, University of Milano-Bicocca, Milan 20126, Lombardy, Italy; Department of Earth and Environmental Sciences, University of Milano-Bicocca, Milan 20126, Lombardy, Italy; Department of Biotechnology and Biosciences, University of Milano-Bicocca, Milan 20126, Lombardy, Italy; Department of Biotechnology and Biosciences, University of Milano-Bicocca, Milan 20126, Lombardy, Italy

**Keywords:** participatory science, environmental microbiome, microbiome education, microbiome sampling, science with society, citizen science

## Abstract

Environmental microbiota are essential yet often overlooked, with urbanization driving microbial diversity loss and diseases of civilization. Public misconceptions, exacerbated by COVID-19, have widened the gap between microbiologists and society, highlighting the need for science-society integration. The *Bicocca Sampling Days* (*BSDs*) model offers a reproducible “citizen science” framework integrating research, education, and public engagement through large-scale microbiome sampling. Tested while assessing environmental microbiomes in different urbanized outdoors, 76 undergraduates participated in four sampling events, collecting 2429 samples in 8 h of effective sampling, achieving over than 303 samples/hour in 29 288.74 m^2^. Manual metadata curation revealed only 0.58% critical errors and no data loss, emphasizing the effectiveness of structured submission forms in ensuring data quality. Educational outcomes, assessed through a validated survey, significant gains in participants’ perceived skills, understanding, and knowledge of microbiome sampling compared to non-participants. The *BSDs* model, including a step-by-step guide, illustrated protocol, and templates, is freely available for replication. Our findings demonstrate that citizen science can rival or complement traditional microbiome research in efficiency, scale, and data quality while broadening accessibility. BSDs offers a scalable tool to advance educational and societal, empower participation, and support informed decision-making.

## Graphical Abstract

**Graphical Abstract:**
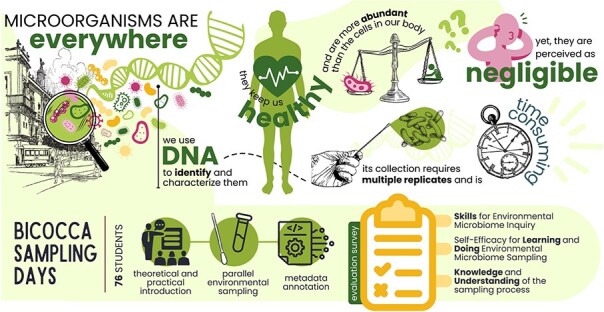

## Introduction

### It's the little things in life

“*We are symbionts on a symbiotic planet, and **if we care to**, we can find symbiosis everywhere*” [1]. Human life is deeply interconnected with the omnipresent and invisible microbial world, yet its evolutionary and health roles are often overlooked.

Since the dawn of life on Earth, microorganisms have shaped its evolution. One widely supported hypothesis suggests that life began ~3.8 billion years ago with ancient microbes inhabiting alkaline hydrothermal vents [2, 3]. Microorganisms henceforth contributed to all evolutionary transitions: the emergence of eukaryotic cell, meiosis, multicellularity, and nervous systems [4]. This symbiotic view of evolution relies on the holobiont concept, which considers organisms and their symbiotic microbiota as an evolutionary unit [5]. Changes in host or microbial genomes affect the hologenome (i.e. the genetic information of the host and its microbiota summed up together), thus driving holobiont evolution and affecting species fitness and development [5].

Humans originated ~200 000 years ago, and have faced multiple adaptations [6]. The most recent is the transition from rural to urban life, which consistently changed the exposome [7], and hence the human microbiota by decreasing diversity and increasing modern-lifestyle-associated microbes. For instance, urban populations tend to exhibit microbiomes enriched in bacterial families associated with high-fat diets, such as *Bacteroidaceae*, *Lachnospiraceae*, and *Rikenellaceae*, while showing a depletion of groups specialized in the degradation of plant polysaccharides, including *Prevotellaceae*, *Paraprevotellaceae*, *Spirochaetaceae*, *Clostridiaceae*, and *Succinivibrionaceae*. [8]. The number of human-associated bacteria is on the same order of magnitude as that of human cells, but they slightly outnumber them (~3.8 × 10^13^ bacterial cells versus 3.0 × 10^13^ human cells) [9], contributing to the maintenance of the physiological functioning of the immune systems, and protection against infectious and noncommunicable diseases [5, 10]. Urbanization has led to the depletion from the environment of microorganisms that co-evolved with humans for hundreds of thousands of years, contributing to the rising immunoregulatory disorders of the last decades [11]. According to the Hygiene Hypothesis, later renamed as the Old Friends Hypothesis, the mismatch between the poorly biodiverse microbiota in our habitats (~75% of Europeans live in urban areas and spend up to 90% of time indoors [12–14]) and our immune system that evolved under high microorganism exposure results in immunoregulatory deficits [11, 15], which are now known as *diseases of civilization* (including but not limited to coronary heart disease, hypertension, obesity, diabetes, and autoimmune disease) [16, 17].

Preserving microbiota biodiversity is evidently fundamental for future health in progressively more urban societies. Yet, human microbiotas are as essential as overlooked. This paradoxical relationship is unsurprising, considering that the social representation of what microorganisms are, meaning their public perception, has not benefited from the last pandemic years [18]. The COVID-19 pandemic amplified the gap between microbiome science and public health policies. It fueled a fear-of-a-single-contagion (that, without considering the interaction dynamics between micro and macro organisms in the host-ecosystem, opposes the holobiont concept [19]) and promoted mandates that, as a result of mixed signals from the scientific community, media, and government institutions, undermined human microbiomes along with human physical and mental health [18, 20]. On one hand, concerns have been raised about the excessive use of antibiotics and the “too much hygiene” practices adopted during the pandemic, due to their potential impact on microbiome dysbiosis and the long-term costs of antimicrobial resistance [21, 22]. On the other hand, the psychological consequences of enforced measures such as quarantine and lockdown have manifested as social isolation, anxiety, depression, sleep disturbances, increased suicide risk, and post-traumatic stress symptoms [23]. Additionally, microorganisms perception was affected: viruses, part of our microbiota, became symbolic of danger and this “COVID-19 effect” reinforced negative perceptions of all microbes [24]. A study of schoolchildren in Buenos Aires demonstrated that post-pandemic children associate microorganisms mostly with viruses while reinforcing their conceptions of microbes as harmful [25].

Understanding how the public perceives microbiology means understanding how common sense forms [26], and how scientific knowledge can inform public health decisions. Since the 1960s, scholars have debated science-society relations. Terms like *Science Literacy* and *Public Understanding of Science* intrinsically imply a public deficit of knowledge or attitudes and omit the necessity of public support for scientific impacts. More recently, the Science-in-Society paradigm overturned deficit attributions and highlighted the lack of trust of scientific practitioners towards the public [26]. These bidirectional deficits also concern the public perception of microbiology and microbiologists’ distrust of the public. How can we deal with disparities between common knowledge and the novel holobiont perspectives while effectively impacting societal health by participating in policy making? We are proposing a model of science-in-society and society-in-science in environmental microbiome studies that aims to improve the microbiology-public relationship by exploiting public skepticism as an educational opportunity and focusing on sharing scopes and increasing impact duration, while passing down scientific methods and rationales rather than merely transmitting facts.

### Open approaches in microbiome research

When the generation of scientific data involves nonexpert volunteers and addresses a specific health or societal issues, the term “citizen science” is commonly used. Providing an exact definition of this term, however, is challenging as it is complicated by variations across countries, contexts, organisations, and stages in the development of citizen science [27]. Here, we use citizen science or, more generally participatory science, to refer to all the different models of public participation in all stages of scientific research [28].

Given their omnipresence and importance to health, microbes naturally invite citizen science. Public interest in their microbial selves has driven many large-scale projects [29]. One of the most renowned examples is the American Gut Project (2012–2017) which involved over 10 000 citizen scientists, allowing the comparison of thousands of human stool microbiomes to environmental samples, showing an unexpected range of beta-diversity and demonstrating that citizen scientist collected samples recapitulate the ones collected by scientists in clinical conditions [30]. The Isala project, which started in 2020, catalogued the vaginal microbiome thanks to the contribution of 3345 Belgian women [31]. Willis *et al.* [32] discovered that the oral microbiome of adolescents is influenced by their lifestyles and the composition of the drinking water they ingest by involving 1500 Spanish teenagers in their study. In the field of environmental microbiome, the Earth Microbiome Project gathered hundreds of researchers from 161 different institutions, and 97 different subprojects generating an unprecedented diversity characterization and providing a framework for data integration [33]. Additionally, the Ocean Sampling Day Consortium, started in 2014, integrates data from more than 190 sites worldwide to study the marine microbiome [34]. Together, these examples demonstrates that public participation in microbiome research is not only possible but expands sampling scales and impacts.

Microbiome studies often face experimental challenges and citizen science, even though requiring specific, additional efforts, helps overcome these hurdles. The collection of microbiome samples involves methods, such as scraping surfaces with swabs or collecting target specimens, which are inherently punctiform. This leads to variability in microbial loads across biological replicates, masking subtle differences in microbial communities [35, 36]. Additionally, the need for a high sample number and replicates is not always compatible with the workforce availability, requiring extended sampling periods. This prolonged time frame can reduce standardization and introduce variability, ultimately impacting both cross-sectional and longitudinal studies [36]. Via citizen science, the power and the potential of microbiome studies are increased by recruiting myriad research members investing little to no funds, reaching vast geographical areas, analyzing huge datasets, or providing high volumes of samples [29]. Although the potential of citizen science is undeniable, achieving meaningful public involvement remains challenging. Participation often remains limited to data collection or data interpretation, or alternatively only to study co-design, leaving contributors with a partial understanding of the research process [28]. In addition, while participant recruitment, community outreach, and science communication require only limited financial resources, these activities demand substantial time, skills, and long-term commitment from researchers. Sustaining citizen science initiatives is further complicated by the lack of dedicated funding mechanisms and by the undervaluation of multidisciplinary projects that integrate participatory approaches with core scientific objectives [37]. The scientific community often perceives, legitimately or not, samples and data collected by nonexperts as less reliable. However, standardized and optimized quality-assessment frameworks, although not yet widely adopted, can significantly improve data credibility [38] and ensure that participatory approaches do not compromise sample collection and handling (contamination avoidance, transportation, and storage) or metadata acquisition. These measures should always be complemented by an efficient ex-ante and continued communication with participants, ensuring clear guidelines protocols are provided [39].

**Figure 1:**
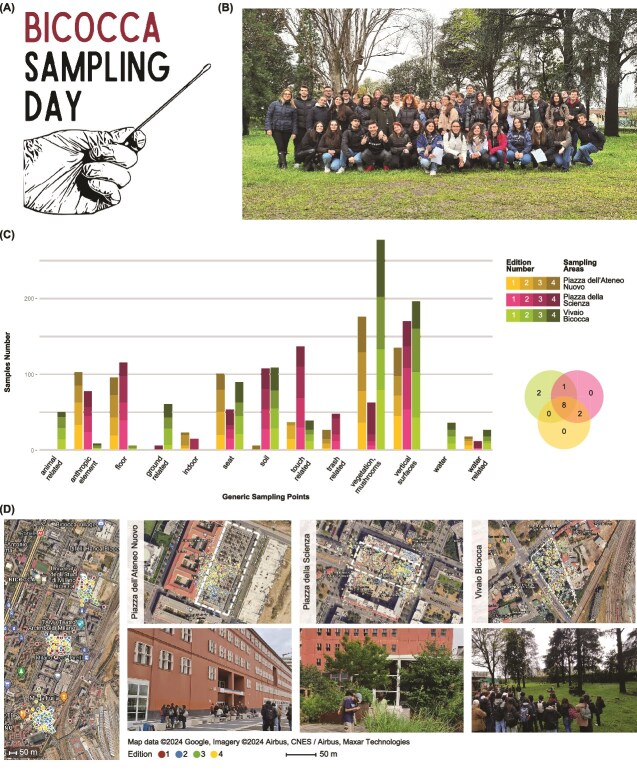
(A) Logo of the *BSDs* initiative; (B) photo of participants and researchers of the last edition of the *BSDs*; (C) bar chart of the generic sampling points by sampling number, on the left and the color legend of sampling areas and edition number, and the Venn diagram of the number of sampling points shared between sampling areas, on the right; (D) map of collected samples. From left to right, the overview of the Bicocca district, the aerial views, and photographs of the three sampling areas (as indicated in the legend, the dots’ color represents the edition samples have been collected in, while the white dotted line outlines the investigated areas). An explorable version of the map is available at https://www.google.com/maps/d/edit?mid=1BkmmK5e1_XUc4f5dda6_HH7W7loxs0c&usp=sharing [40].

To meet these needs, we designed a scalable, reproducible approach to investigating specific environmental microbiome-related questions. We also engaged new audiences through informal science education and tested a new participatory science model, the Bicocca Sampling Days (*BSDs*) model (**Graphical abstract,**  Fig. 1A and B). Over a year, 76 students participated in four sampling events across three outdoor areas of the Bicocca district, an urban area in northern Milan, chosen for varying urbanization levels. Volunteer participants collected samples (soil, water, and urban, and vegetation surfaces) driven by the hypothesis that microbial communities are influenced by the urbanization, contributing to a dataset intended for peer-reviewed publication. Participants actively contributed to model design and testing, and educational impacts have been assessed.

## Materials and methods

### Student recruitment and communication

Undergraduate students from the University of Milano-Bicocca were recruited through academic outreach channels, including mailing lists, social networks, and flyers. Participation was on voluntary basis, and the only inclusion criteria was being a student. Depending on their participation in the *BSDs*, the participants and nonparticipants groups have been established. A dedicated web platform hosted registration and provided project details, including a schedule and FAQs, and is accessible at the following link (https://www.eventbrite.it/cc/bicocca-sampling-days-2568289?_gl=1*68v8oj*_up*MQ..*_ga*MjQ3NTE2NzIwLjE3MzY1ODk3MTQ.*_ga_TQVES5V6SH*MTczNjU4OTcxMy4xLjAuMTczNjU4OTcxMy4wLjAuMA. [41]). A consistent visual identity was developed supported communication efforts to ensure clear and recognizable branding. For each new edition of the project, participants from previous events were encouraged return, fostering continuity and community.

### Student-science event

Each of the four BSDs (held on 22 June 2023, 29 September 2023, 20 December 2023, and 27 March 2024, covering all seasons) followed the same 4-h schedule. Participants were welcomed at the registration desk based in Vivaio Bicocca (see the “Sampling Areas” paragraph), where they could request the certificate of attendance and sign the image release forms (see “Metadata Annotation”). Participants attended a 1-h introduction to general concepts of microbiome research and a practical demonstration of the sampling procedure to avoid possible ambiguities in protocol interpretation. The covered core concepts were derived from the Microbiology Concept Inventory (MCI) fundamental statements [42] and integrated for the specific aims of the campaign, and are listed in the Additional File 1. Participants were divided into nine groups and equipped with the printed protocol, sample checklist, and QR code linked to the metadata submission form. Sampling lasted about 2 h. Supervisors were constantly available for questions and guidance and were responsible for registering samples returned by participants to the cooler, where samples were provisionally stored. Finally, all participants and supervisors gathered to discuss the sampling, collect feedback, and share an informal, convivial moment over some food.

### Sampling areas

Sampling occurred in Milan’s Bicocca district (Italy), near the university campus. Three outdoor sampling areas, with different degrees of urbanization, were selected: (i) *Vivaio Bicocca*, a green urban nursery, and setting for participatory science and scientific research on functional biodiversity. It hosts an apiary, a pond, benches, and wooden stools for open-air lectures. (ii) *Piazza della Scienza* (literally, Science Square), the square overlooking the buildings of the scientific department. It is mainly cemented, but also includes two small gardens (see Fig. 1D). Considering the surface proportion of built environments (including all artificial structures) in each sampling area, *Vivaio Bicocca* comprises ~1% built surfaces, *Piazza della Scienza* 80%, and *Piazza dell’Ateneo Nuovo* 100%. After the first summer edition, the square was restructured, thus the participants sampled an ongoing construction site. (iii) *Piazza dell’Ateneo Nuovo*, the square in front of the main building, is completely cemented and few trees emerge from the concrete. For each sampling area, surface areas were measured using the *measure distance and area* feature on Google Earth.

### Sample collection and storage

Sampling materials (swabs, tubes, and bottles) were prepared in advance in numbered triplicates (nA, nB, and nC) to facilitate triplicate collection. Sampling was guided by a paper checklist ensuring that a batch of samples is collected in all editions and without overlapping between participant groups. The checklists allow participants to write each sample number on the corresponding item of the checklist, but also to include user-defined sampling points. Participants used sterile polyester Copan swabs (pre-wetted with Milli-Q water) for surfaces, and sterile 50 ml tubes/1 L bottles for soil and water. Surface and soil samples were kept on ice and stored at −20°C within 4 h. Water samples were kept at 4°C until filtration, which occurred in the next 24 h. Three 250 ml replicates from each bottle were filtered with sequential 8.0 μm and 0.22 μm sterile filter membranes to avoid filter clogging. Filters have then been stored at −20°C.

### Metadata annotation

Participants submitted metadata with their phones using a browser-based form (Additional file 1) designed with the open-source suite KoboToolbox [43]. Each replicate was photographed and described indicating its sampling area (Vivaio Bicocca, Piazza dell’Ateneo Nuovo, Piazza della Scienza), sample type (soil, water, and surface), surface type (when applicable), coordinates, and sampling date and time. Metadata annotation was required for each sample replicate as a strategy to minimize random errors. Within each group, multiple participants were responsible for metadata annotation; however, no systematic control over the exact number of annotators was implemented. Details on the weather (mean temperature, relative humidity, atmospheric phenomena, and weather conditions) were downloaded from a public archive available at https://www.ilmeteo.it/portale/archivio-meteo/Milano [44].

### Metadata curation

Submitted metadata were downloaded and manually curated for consistency across replicates, photos, and descriptions. Additionally, standardized sampling points have been coded in three hierarchical levels: generic (13 categories), specific (99 categories), and intermediate (29 categories) to simplify sample overview.

### Educational impacts evaluation

An evaluation survey was used to assess the effectiveness of the *BSDs* in increasing participants’ knowledge, understanding, and awareness of microbiome-oriented research. Survey’s design is based on the “User’s Guide for Evaluating Learning Outcomes in Citizen Science” of the Cornell Lab of Ornithology [39], and the Citizen Science Evaluation Framework [45] available on the EU Citizen Science Platform. All data collected from surveys were managed in accordance with the General Data Protection Regulation (EU) 2016/679 (GDPR) [46]. Participants were informed about the purpose of the study, data processing procedures, and their rights regarding data protection. Data were anonymized to ensure confidentiality. To quantitatively evaluate the educational impacts of the *BSDs*, we administer participants and nonparticipants a survey designed with standard and customized scales. The survey was written in Italian to match the respondents’ mother tongue, but we provide an integral, bilingual (Italian and English) version in Additional file 1. This aims to assess participants’ perceived science inquiry skills, and all responders’ self-efficacy for learning and doing environmental microbiome sampling, the level of understanding of the “Environmental Microbiome Sampling” process, and its knowledge.

- **Attitudes toward science and the environment**
  Assessment of respondents’ attitudes toward Science and the Environment was carried out following the protocol of Brossard *et al.* (2005) [47]. We used the indicated MATOSS and NEP/humans-with-nature subscales to limit the overall survey length, as Cornell Lab of Ornithology’s User’s Guide [39] suggested.
- **Skills for environmental microbiome inquiry**
  Participants’ perception of their abilities to pursue the environmental microbiome sampling before and after attending the *BSDs* was estimated with the Skills for Science Inquiry retroactive customizable scale [48]. It consists of 12 five-point Likert questions that measure respondents' perceived science inquiry skills in the context of a specific project. The scale is administered in two versions, acting as a retrospective pre- post-test.
  We reduced the item number to 8, to maintain coherence with the *BSDs*’ structure and contents. Each question has five answering options (Strongly Disagree, Disagree, Neutral, Agree, Strongly Agree) and scoring was assessed according to Phillips *et al.* (2017) [48]. The full scale has internal consistency (Cronbach’s alpha) equal to 0.912. Standard calculation of Cronbach’s alpha, using the *ltm* R package [49] of our 8-item version of the scale's internal reliability resulted in alpha = 0.953. We also used the *psych* R package [50] to compute Revelle’s omega total coefficient estimating the covariances between items with a polychoric covariance matrix rather than with a Pearson covariance matrix to meet the normal distribution assumption as suggested by McNeish (2018) [51]. Using the script provided in Additional file 2, we obtain Revelle’s omega total coefficient = 0.99. To test whether starting levels of perceived skills affect the *current* scores, we grouped the participants into those with low (below the midpoint) *retrospective* perceived skills and those with high (above the midpoint) *retrospective* perceived skills.
- **Self-efficacy for learning and doing environmental microbiome sampling**
  We used this scale to measure respondents’ confidence in learning and doing microbiome sampling-related topics and activities. It comprises eight 5-point (Strongly Disagree, Disagree, Neutral, Agree, Strongly Agree) Likert questions (4 centered on evaluating learning, and 4 focused on doing) whose scores assess perceived competence. Customization, administration, and scoring were performed as indicated in Porticella *et al.* (2017) [52]. The reported Cronbach’s alpha equals 0.92 [52].
- **Understanding of the “environmental microbiome sampling” process**
  The comprehension of the environmental microbiome sampling in participants and nonparticipants was assessed with a closed-ended question and a consequent open-ended one. This scale structure was previously used by Brossard *et al.* (2005) 47 and the National Science Board (1996) [53] to evaluate the understanding of the term “scientific study”. We adapted this scale to change the evaluated construct. The first question asks respondents to classify their level of understanding as “clear”, “general”, or “little”. People who declared to have a “general” or “clear” comprehension of “environmental microbiome sampling” were then asked to describe the term in their own words. To score the responses, we identified a priori 8 elements that would comprehensively define the “environmental microbiome sampling”. For each element included in the responders’ definition, we added 1 point, thus defining 0 to 8 scale scores.
  Using the *psych* R package [50] and the script provided in Additional file 2 we obtain Revelle’s omega total coefficient equal to 0.89 (polychoric covariance matrix). Survey submissions with missing values were excluded from the internal reliability testing.
- **Knowledge of the “Environmental Microbiome Sampling” process**
  To evaluate the impact of the *BSDs* on the individual knowledge of environmental microbiome sampling, we constructed a 10-item scale of true/false questions following the model of Brossard *et al.* (2005) [47] that instead aimed to assess knowledge of bird biology. Each item contributed equally to the scale score (1 point for each item answered correctly for a maximum total score of 10). Following the R script provided in Additional file 2 and using the *psych* package, we obtained Revelle’s omega total coefficient (polychoric covariance matrix) of 0.84.

**Figure 2:**
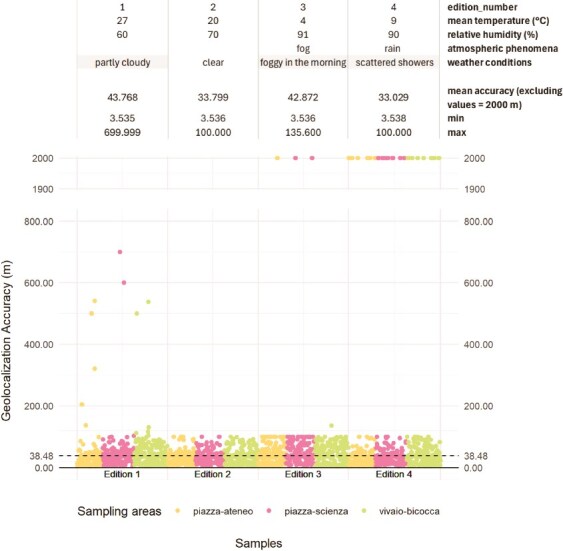
Dot plot of samples’ geolocation accuracy by sampling area and edition. For each edition, temperature, relative humidity, atmospheric condition, weather condition, and geolocation mean, minimum, and maximum values are indicated.

## Results

### The Bicocca sampling days model allows mass, rapid, efficient, and parallel environmental sampling

Participants collected 2429 samples in four editions (that equals 8 h of effective sampling activity) rating more than 303 samples/hour in a total area of 29 288.74 m^2^, a pace that standard sampling practices cannot match. Sample types included 2177 swabbed built surfaces (including but not limited to tree trunks and leaves, floors, walls, benches, and handrails), 216 soil samples, and 36 water samples. The 13 generic sampling point classifications are shown in Fig. 1C by sample number. The checklists guided sampling activities efficiently, with 8 generic sampling point categories shared across all sampling areas. Area-specific sampling types included water and animal-related categories exclusive to the nursery Vivaio Bicocca, and indoor/trash-related ones exclusive to the squares. Intermediate categories are listed in Table S1 in Additional file 1; specific points are available in the metadata (Additional file 3).

### Geolocation accuracy was generally reliable, though variable

Samples were geotagged using participants’ mobile GPS sensors via the KoboToolbox point “GPS” question that returns longitude, latitude, altitude, and accuracy values (Fig. 1C). Geolocation accuracy depends on the quality of the GPS sensor, the cloud coverage, and structures obstructing the view of the sky [54]. Some samples in editions 3 and 4 reached inaccuracies up to 2000 m. Excluding these outliers, the mean geolocation accuracy is 38.48 m (median value 35.00 m). Our records were consistent within the event edition, but the four editions differ from each other. This was attributed to device variability and weather conditions, as shown in Fig. 2, excluding the influence of sky visibility or buildings' presence since no peculiar differences are observed among sampling areas.

### Student-annotated metadata are accurate and reliable

Students annotated metadata with their phones, filling out the metadata annotation form (see Metadata Annotation paragraph of Materials and Methods). To assess the reliability of student-annotated metadata, we manually curated all entries and documented errors. Table 1 summarizes the number, type, and examples of inconsistencies found in metadata fields such as sample name, sampling area, and sample type. Categorizing these errors helps evaluate their impact on data quality. The most severe error, misspelling of sample names or replicates, can compromise correct association of sample data. Among the 2429 samples, only 14 had incorrectly named samples, resulting in a 0.58% error rate. The most frequent errors involved sample replicates, either unclearly labeled (1.85%) or containing typos (1.52%). While these errors do not affect the sample information itself, they require extra time for correction. Error frequency is closely tied to the metadata form design: open-ended questions intrinsically introduce more typos, whereas multiple-choice questions reduce them. Nonetheless, all errors were resolved through cross-checking checklists, geolocation data, photos, and digital submissions. No sample data were lost.

**Table 1:** Summary of the errors recorded during metadata manual curation by metadata column. Examples, numbers, and rates of errors are shown.

**Metadata column**	**Error type**	**BSD edition**				**Total**	**Error rate**
		**BSD1**	**BSD2**	**BSD3**	**BSD4**		
BSD_sample_number_replicate original name submitted by participants (ex: *153A*)	Missing replicate	1	4	3	5	13	0.54%
	(ex: *185A, 185C* instead of *185A, 185B, 185C*)						
	Implicit replicates	-	23	-	22	45	1.85%
	(ex: *185* instead of *185A, 185B, 185C*)						
	Misspelled sample name	-	-	3	2	14	0.58%
	(ex: ***158**A**, 158**B, **158**C* instead of *185A, 185B, 185C*)						
	(ex: *158A, **185**B, 158C* instead of *158A, 158B, 158C*)	-	5	4	-		
	Misspelled sample replicate name	9	7	10	11	37	1.52%
	(ex: *158A, 158**C**, 158**C*** instead of *185A, 185B, 185C* or *136**F*** instead of *136A*)						
	Replicate metadata submitted twice	1	3	2	1	7	0.29%
	(ex: *158A, 158**B**, 158**B**, 158C* instead of *185A, 185B, 185C*)						
sampling_area sampling area submitted by participants (ex: *Vivaio Bicocca*, *Piazza dell'Ateneo Nuovo*, *Piazza della Scienza*)	Misplaced sample replicate	3	-	3	1	7	0.29%
	(ex: *piazza-ateneo* instead of *piazza-scienza*)						
	Double selection	2	1	1	1	5	0.21%
	(ex: *piazza-scienza piazza-ateneo* instead of *piazza-scienza*)						
sample_type sample type submitted by participants (ex: *water*, *soil*, *swab*)	Wrongly described sample replicate	1	3	-	-	4	0.16%
	(ex: *swab* instead of *water*)						
	Double selection	5	2	4	3	14	0.58%
	(ex: *swab water* instead of *water*)						

### The Bicocca sampling days model was co-designed by participants and researchers

The joint efforts of researchers and students led to the development of a participatory science educational model for microbiome research. The model aims to make participatory microbiome research scalable, reproducible, and educationally effective. It was co-designed during iterative cycles of planning, implementation, feedback, and evaluation. Structurally, the BSDs model consists of three main components:

- **A standardized scientific protocol**, including detailed sampling procedures, metadata submission forms, checklists, and contamination-avoidance guidelines ensuring that nonexpert participants can collect reliable and comparable data.
- **An educational module**, providing theoretical background, hands-on demonstrations, and structured reflection moments before and after fieldwork to enhance conceptual understanding and inquiry skills.
- **A participatory process** in which volunteers contribute not only as sample collectors but also as co-designers of the sampling strategy, and co-producers of knowledge in the interpretation of the generated data.

The *BSDs* model is scalable, reproducible, and customizable. To facilitate the adoption of participatory sampling in microbiological research, we have created and provided detailed documentation. A step-by-step guide to designing a participatory sampling campaign (from defining the research question to outlining the event and evaluating its educational impact) is available. Additionally, we provide an illustrated sampling protocol, the checklists used during the *BSDs*, and templates for the metadata submission form and the evaluation survey. All resources are available in Additional file 1 and deposited on Open Science Framework (see **Data availability**).

### The Bicocca sampling days participatory microbiome sampling model increases participants’ self-competence, knowledge, and awareness

We administered the evaluation survey to 71 respondents, all enrolled in scientific courses (66 from biology-related degrees, and 5 from chemical and environmental sciences fields). The entries and scoring of each respondent are available in Additional file 4. To support the homogeneity of their backgrounds, we tested all respondents’ attitudes toward Science and Human-with-Nature concerns. Both participants and nonparticipants had comparable levels of pro-science attitudes (T-test, *P* = .1163) and attitudes against human action on the environment (Mann–Whitney-Wilcoxon Test, *P* = .3585) (Additional file 1).

- **Participants gain perceived abilities to pursue environmental microbiome sampling after attending the *BSDs***

**Figure 3:**
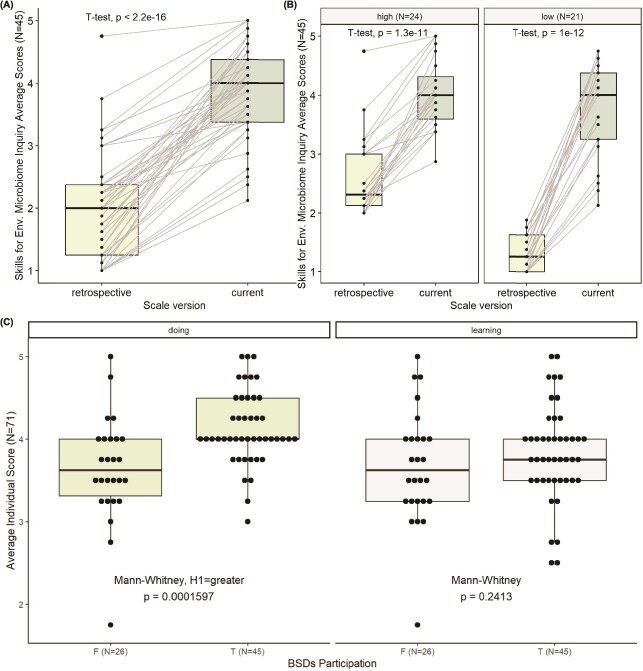
(A) Paired average individual scores of *BSDs*’ participants from the skills for environmental microbiome inquiry scale current and retrospective versions; (B) skills for environmental microbiome inquiry paired scores for participants divided into low and high initial levels of perceived abilities; (C) average individual scores for the self-efficacy for learning and doing environmental microbiome sampling subscales of participants and nonparticipants. Statistical tests and *P*-values are indicated in each panel.

The *Skills for Environmental Microbiome Inquiry* scale was conditionally administered only to respondents who previously participated in the *BSDs*. Out of the 76 total *BSDs* participants, 45 completed this scale. The comparison of entry frequencies and scale mean scores between *current* and *retrospective* scale versions is presented in Table S2 in Additional file 1. *Current* scores are significantly higher than *retrospective* ones (paired T-test, *P* < 2.2e-16) (Fig. 3A), meaning that participating in the *BSDs* positively increases abilities to collect microbiome samples, annotate metadata, plan and carry out sampling campaigns, and communicate related information. Participants were grouped into high and low *retrospective* perceived skills groups. While both the groups show an increase in perceived skills (paired T-test, *high*: *P* = 1.3e-11, *low*: *P* = 1e-12) (Fig. 3B), the differences in scores (*current—retroactive*) of the “low” group are significantly higher than the “high” ones (T-test, *P* = 6.903e-06).

- **Participants have stronger self-efficacy for doing microbiome sampling-related tasks, while all respondents have comparable levels of self-efficacy for learning**

We evaluated the self-efficacy for learning and doing environmental microbiome sampling subscales separately, driven by the expectation that being our sample composed of science students, they all might have high confidence in learning scientific topics of any kind. The mean scores of the two subscales are shown in Fig. 3C for both the participants and nonparticipants groups. As expected, the mean scores for the scale evaluating the self-efficacy for learning microbiome sampling-related tasks do not differ significantly between participants and nonparticipants (Mann–Whitney test, *P* = .2413) (Table S3 in Additional file 1, Fig. 3C). On the other hand, scores related to the self-efficacy for doing microbiome sampling are significantly higher for participants when compared to non participants’ scores (Mann–Whitney test, *P* = .0001) (Table S3, Fig. 3C). Students who attended the *BSDs* feel more competent than their colleagues in performing all the tasks required to sample the environmental microbiome.

- **Students who participated in the *BSDs* understand and know better the environmental microbiome sampling process than students who did not.**

**Figure 4:**
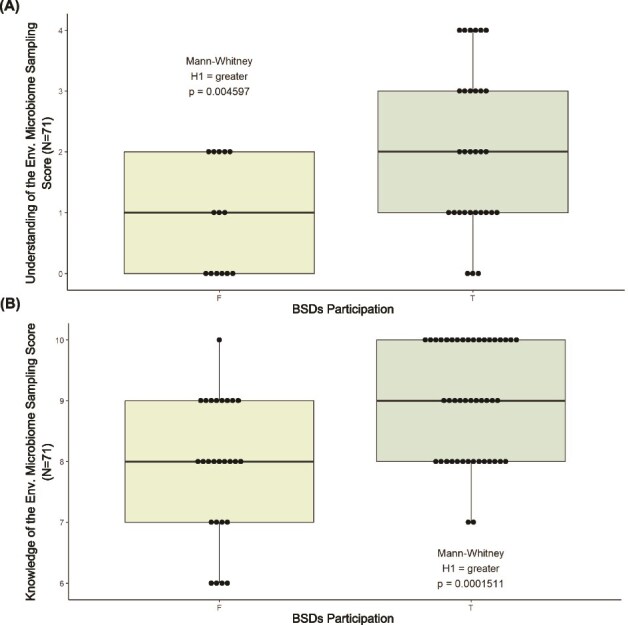
Comparison of the average individual scores for the *understanding* (A) and *knowledge* (B) *of the environmental microbiome sampling process* scales of participants and nonparticipants.

We asked all respondents to define their level of understanding of the sampling process as “clear”, “general” or “little”. The ones who responded “clear” or “general” were nonmandatorily asked to define it in their own words. Only 45 out of 71 total respondents decided to write down their definitions. Since the missing answers can be due to a lack of dedication rather than the incapacity to formulate a definition, we did not consider them as 0-point answers but excluded them from the statistical summary shown in Table S4 in Additional file 1. More than 93% of all respondents claimed to have a “clear” or “general” understanding of the term “environmental microbiome sampling”. According to the number of elements included in the respondent’s definition, their total scores range from 0 to 8 (all elements contribute to the scale score equally, 1 point each). Figure 4A shows that participants' mean scale score (2.06, SD = 1.31) is significantly higher than non participants’ (Mann–Whitney, *P* = .004597). In contrast with respondents’ perception of their comprehension level, there is no difference between those who claimed to have a “general” sense and those who declared to have a “clear” understanding of the term in both the participants (Mann–Whitney, *P* = .4271) and nonparticipants groups (Mann–Whitney, *P* = .3413). Evaluating significant differences within the single elements scoring, elements 1, 2, 4, 5, and 6 significantly differ between participants and nonparticipants (Mann–Whitney, 1: *P* = .03689, 2: *P* = .03689, 4: *P* = 7.17e-05, 5: *P* = 2.985e-06, 6: *P* = .005962). Participating in the *BSDs* has given students specific insights on avoiding user contamination during the sampling, annotating sample metadata, using specific tools, focusing attention on microorganisms within a particular environment, and aiming at collecting their DNA that otherwise students only sometimes acquire, thus *BSDs* participants have a higher understanding of the microbiome sampling process.

Concerning their knowledge, respondents answered a 10-item true/false scale (each correctly answered item adds a point to the overall score). After participating in the *BSDs*, participants' mean score for knowledge of the environmental microbiome sampling process nearly exceeded nonparticipants’ mean score by more than one full point, a statistically significant result (Mann–Whitney test, *P* = .00015) (Table S4 in Additional file 1, Fig. 4B). In detail, considering each item separately, we identified items 2, 3, and 10 as the ones with statistically significant differences in mean scores between participants and nonparticipants (Mann–Whitney, 2: *P* = .004982, 3: *P* = 5.64e-05, 4: *P* = .005462). Zooming in on the scale items allows us to single out the microbiome investigation-related topics students do not commonly learn during their biology degrees. We found out that nonparticipants are less prone to consider metadata compilation as part of the sampling process, the correct sample storage, and the advantages of the metagenomic approach compared to traditional microbiology. Administering these scales to the general public or groups with no scientific background is expected to result in stronger differences in more and different elements and items.

## Discussion

Citizen science or, in this case, student science projects like *BSDs* (meaning a participatory study dedicated only to students), require multidisciplinary skills and yield multidisciplinary outputs. However, traditional scientific training often lacks the socio-educational skills needed for participatory research, and limited interdisciplinary funding hampers scalability 37. Despite these challenges and the organizational effort required for coordination and data management, we argue that the benefits of involving nonexperts in scientific research far outweigh the associated costs. The *BSDs* model addresses this gap by providing structured resources that enable their application without requiring extensive retraining. It is also the only participatory microbiome-related project to our knowledge that includes a validated educational evaluation framework.

*BSDs* simultaneously produce scientific and educational outcomes, benefiting researchers, participants, and communities. Defined as data collection-aimed, curriculum-based, and co-created [28, 55] the model demonstrates how large-scale environmental microbiome sampling can be efficiently achieved through participatory approaches. Fieldwork can be laborious and time-consuming. In a single edition, more than 600 environmental microbiome samples were collected by participants in an area of almost 30 000 m^2^ to be covered by foot, and metadata annotated—work completed in just 2 h. Considering the same individual sampling rate (4 samples per person per hour over an area of 30 000 m^2^), we estimated that achieving the same yield from a single edition would require more than 150 h of sampling by a single researcher, or over 600 h to collect all 2429 samples obtained across editions. Metadata accuracy is the requisite for meaningful analysis, and a common concern in projects led by individuals without formal scientific training [26, 55, 56]. The guided format of the *BSDs* model balances participants’ independence and initiative allowing for participant-driven observations, with the trustworthiness of precise metadata and standardized data input. Metadata error rates evaluation revealed no sample loss due to missing information, and any inaccuracies were recoverable through cross-referencing metadata fields. The only error type that could not have been detected are systematic errors in all replicates of a sample. When designing the metadata collection form, researchers should consider that the more restrictive it is (preferential use of closed-ended questions), the less manual curation is required afterward but the less additional participant-derived information is collected. Importantly, incorporating photographic documentation in the metadata collection process provides additional context, enabling researchers to verify sampling locations, surface types, and sample identifiers, and encouraging participants to follow instructions more carefully and engage with the sampling process. Future iterations should incorporate metadata cross-checking, in which more than one participant annotates the same sample replicate to identify inconsistencies, alongside automated error-detection algorithms in digital forms to improve data reliability and minimize manual correction time. One additional consideration for future iterations of the *BSDs* is the accuracy of GPS coordinates collected via participants' mobile devices. While precise location data was not essential for this study, meteorological conditions and device variability can affect GPS accuracy. Providing standardized GPS devices could improve data consistency. Additionally, providing devices prevents participants’ need to use personal phones and possible privacy concerns. Nevertheless, no personal or device data were collected while using the metadata submission form. The approach proposed during the BSDs entails a limited environmental applicability of the online metadata collection form that could not be reachable, or GPS coordinates recorded in remote natural environments such as forests or mountains.

Participation in data collection can change awareness, understanding, and knowledge of microbiome-related topics [57], but may not be enough to shift attitudes [55]. The co-creation nature of the *BSDs* model addresses this limitation by involving participants in the scientific process beyond mere sample collection. Each group selected autonomously approximately a dozen sampling points sampled in replicates, about a hundred total per edition, encouraging ownership of experimental design. The creativity and intelligence of all trained participants, aligned with the research question, enriched the sampling design with their nonstereotypical and field-based perspectives including samples such as the tram tracks, bike parking, and stoplight buttons. It is also important to acknowledge a potential collateral limitation of student-driven samples: some sampling points may be unique or underrepresented, leading to sparse data that can restrict comparative analyses within the broader dataset. The participants’ inputs reflected strong alignment with urban microbiome research strategies used in global initiatives like the MetaSUB Consortium [58], proving their relevance. Experiential learning foresees a reciprocal influence between learners and the learning context [59]. Participants played an active role in shaping the *BSDs* model itself, and those involved in at least three events are credited as co-authors of this manuscript. This deep engagement increases participants’ interest and skills and specifically enhances their knowledge of the scientific process [28]. Co-authors had access to the generated data during manuscript writing, but the published results will be shared with all participants, as a form of restitution and recognition of their contribution.

Lastly, we defined our model as curriculum-based because it is specifically designed to investigate the research question while fostering educational goals for youth. Designed for undergraduates, *BSDs* provide young people with a unique understanding of microbiome research, investing in the development of our broader community and in long-term social changes towards microbiology [27, 55, 60]. While students in life sciences programs often receive theoretical microbiology training, they rarely gain hands-on experience with current concepts. The integration of new scientific concepts, such as the recent microbiome trend, into education often lags behind their rapid emergence (didactic transposition delay) [18, 61]. This delay means that soon-to-be scientists frequently receive formal training i.e. incomplete from the outset. Efforts to define the fundamental microbiology concepts to be taught in degree programs are already establishing frameworks and best practices to measure learning outcomes [62]. The American Society for Microbiology Curriculum Guidelines defined the new microbiology concept inventory [42] which we complied with defining the *BSDs* theoretical structure. Beyond factual knowledge, the *BSDs* model fosters research practices and uncertainty, the problem of science reproducibility, and the complexity of the controversies over the roles of microorganisms. These concepts and attitudes are only perceivable through active participation in scientific research. Campaign for Learning’s definition of learning states that “*Learning is a process of active engagement with experience*” [63], thus it must be intended as a process and not as an event [64].

The *BSDs* model considers learning in all its domains: cognitive (knowledge application and process evaluation), psychomotor (experience-based adaptation), affective (attitudinal engagement) [64, 65], and social (contextualized collaborative learning) [66, 67]. Rooted in experiential learning theory, it focuses on out-of-the-classroom science methods and processes. Outdoor teaching environments, such as the areas investigated during the *BSDs*, allow teaching science in new, experience-based ways that enrich the educational process [65] and address the related problems more efficiently [60]. Nature engagement and microbial knowledge promote positive attitudes towards the microbiota 24, while outdoors connects students with the changing society 68 and the environment. While fieldwork is a recognized contributor to professional development in medicine [69], its limited use in bioscience education is concerning [13, 68], despite being a rewarding, essential learning opportunity [70, 71]. The *BSDs* model addresses this gap by offering structured fieldwork that builds long-term positive attitudes towards the microbiota, microbiological inquiry and procedural skills, knowledge, and understanding. The evaluation survey assessed not only notions but also the feeling of learning and doing scientific research and the perceived ability to investigate environmental microbiomes. Results show that active participation increases participants’ microbiome knowledge, their sampling skills and confidence (especially among those with low initial perceived skills), and their understanding. One limitation of the model is that, relying primarily on self-reported measures, it does not evaluate changes in actual skills or assess the relation between perceived and actual skills.

Having assessed participant impact, we can speculate on the broader community implications. According to Pierre Clément’s KVP model [72], individuals form conceptions through the interplay of Knowledge (K), Values (V), and Practices (P). *BSDs* integrate up-to-date scientific literature-derived concepts (Knowledge) with critical thinking, scientific responsibility, and research attitudes (Practices). Additionally, people’s perception and behaviors are associated with their choices and beliefs [52], defining the *BSDs* as an instrument to acquire and develop individual opinions and attitudes toward microbiology (Values). This suggests that the model could be equally effective, or even more so, when applied to the general public. However, its educational impact cannot be generalized without testing it in different cohorts and adapting the model according to the participants’ cultural background, language and settings.

Compared with existing citizen science initiatives, the BSDs model uniquely combines structured and individually supervised training with standardized environmental microbiome sampling and metadata annotation, while providing an immediate educational return for participants. Most well-established microbiome citizen science programmes, such as The American Gut Project, the Isala Project and the Earth Microbiome Project, are primarily contributory: they are fully designed by scientists, and citizens mainly contribute samples or data to address predefined research questions and expand public engagement. In contrast, the BSDs introduce a co-creation component, actively involving participants in the refinement of protocols and in the overall research process, thereby testing and validating a new participatory science framework [28, 73]. Furthermore, unlike large-scale human microbiome initiatives, the BSDs avoid major ethical and logistical constraints linked to the collection of human samples, which facilitates scalability to schools and broader communities, although applications have so far remained geographically limited. Scaling the *BSDs* to schools and the general public could amplify its societal value, moving from student science to science-with-society. Strengthening public understanding of microorganisms supports informed policymaking and empowers communities to tackle microbiome-related challenges [62]. For example, acknowledging the health implications of microorganisms present in urban environments is crucial for sustainable urban rehabilitation planning. Informed practitioners could integrate this perspective, ensuring that policies and urban design strategies incorporate microbiome-informed principles and infrastructures [74, 75].

## Conclusion

In conclusion, the *BSDs* model demonstrates that participatory science effectively combines rigorous environmental microbiome research with educational and societal gains. Future applications should expand the model’s reach, engage diverse groups and embed it in formal education. The *BSDs* model can be adapted to other microbiome fields (e.g. water, plant, or insect microbiomes) that do not involve invasive sampling of humans or animals, which could pose risks to participants or subjects and require ethical approval.

To maximize the impact of citizen science projects, funding bodies should prioritize interdisciplinary research initiatives that bridge the gap between scientific research and public engagement. Further, scientists must continue to advocate for participatory research as a means to democratize science, enhance public understanding of complex topics, and ensure that scientific advancements translate into meaningful societal benefits. We believe participatory research represents a necessary evolution in environmental microbiology, addressing the current disconnect between scientific knowledge and public awareness. The success of the *BSDs* demonstrates that such models can effectively contribute to both scientific progress and individual empowerment, ultimately promoting a more inclusive and informed approach to science.

## Supplementary Material

Additional_file_1_ycaf220

Additional_file_2_ycaf220

Additional_file_3_ycaf220

Additional_file_4_ycaf220

## Data Availability

The samples’ metadata, questionnaire responses, and freely available and reproducible resources are deposited in a project on Open Science Framework (OSF) and can be accessed at https://osf.io/yje5w/?view_only=abcaf12523b94cf98e72a2cd048444f6 for reuse under license CC BY-NC 4.0.
