# Supplementary material for: The Bicocca sampling days model: a participatory citizen science approach to environmental microbiome research and education
Source: ISME Commun. 2025 Nov 22;5(1):ycaf220. doi: 10.1093/ismeco/ycaf220 (PMC12704432; doi:10.1093/ismeco/ycaf220)
Supplement: Additional_file_1_ycaf220 [file additional_file_1_ycaf220.pdf]

Additional file 1 for
The Bicocca Sampling Days (BSDs) Model: A Participatory Science Approach to Environmental Microbiome Research and Education

Giulia Ghisleni, Sara Fumagalli, Alice Armanni, Asia Rosatelli, Andrea Franzetti, Maurizio Casiraghi, Yodit M. Bacchi, Cristian Barillari, Denyse A. Battista, Michela Benocci, Aurora Brunelli, Federica Cammarano, Giorgio Bovolini, Fabio Capuano, Noemi Bulla, Althea Colombo, Laura Colombo, Luca Corneo, Davide Evangelista, Piero Giorgetti, Giada Marin, Alessia Meziu, Mattia Riva, Davide Rizzo, Simone Romano, Stefano Ronchi, Riccardo Rossi, Roberta Volpi, Marta Zanotti, Marco Zenaro, Antonia Bruno

Giulia Ghisleni
Email: giulia.ghisleni@unimib.it

This PDF file includes:

Supporting text
SI: results
SI: materials and methods
Ready-to-use Resources:
- Metadata submission form template
- Bilingual evaluation survey
- Step-by-step guide for participatory environmental microbiome campaigns
- Illustrated sampling protocol
- Example checklists for environmental microbiome sampling

Figure S1
Tables S1 to S5
Additional File References

Supporting Text

Results: The *BSDs* participatory microbiome sampling model increases participants' self-competence, knowledge, and awareness – All respondents have comparable attitudes toward science and the environment.

All respondents' attitudes toward Science and Human-with-Nature concerns were tested. The Modified version of the Attitude Toward Science Scale (MATOSS) by Brossard *et al.* (2005) (2) is a 4 five-point Likert questions and was used to measure pro (max score +8) or anti attitudes toward science (max score -8). Attitudes toward the environment were assessed with a subscale of the New Environmental Paradigm (NEP) scale (2, 3) including 4 five-point Likert questions evaluating the perception of how human activities are altering the ecosystems (pro max score +8, anti max score -8).

Both participants and non-participants had comparable levels of pro-science attitudes (T-test, p-value = 0.1163) (**Figure S1a**) and attitudes against human action on the environment (Mann-Whitney-Wilcoxon Test, p-value = 0.3585) (**Figure S1b**). Summary of the submissions to the scales, items and their scoring are shown in **Table S5** for the participants and non-participants groups. Scoring for Item 3 and Item 4 of the MATOSS scale has been reversed (Strongly Agree = 2, Agree = 1, Neutral = 0, Disagree = -1, Strongly Disagree = -2).

Material and Methods: Student-Science event - core concepts of the theoretical lesson

The core concepts covered during the theoretical lesson and practical demonstration of each edition of the *BSDs* were derived from the Microbiology Concept Inventory (MCI) fundamental statements (1) and integrated for the specific aims of the campaign. They are listed below:

- Microorganisms are ubiquitous and live in diverse and dynamic ecosystems (1)
- Microorganisms, cellular and viral, can interact with both human and non-human hosts in beneficial, neutral, or detrimental ways (1)
- Microbes are essential for life as we know it and the processes that support life (1)
- Metagenomics is more informative than isolate cultures for microbiome characterization
- Human impact on the environment influences the evolution of microorganisms (e.g., emerging diseases and the selection of antibiotic resistance) (1) and vice versa
- Swabs, collection tubes, and bottles are the tools used to sample environmental microbiomes (practically demonstrated)
- Sample replicates guarantee robust and reliable data
- Since microorganisms are ubiquitous, sampling and sample processing must be performed with caution avoiding user-derived or cross-sample contamination
- Collected samples must be conserved at 4°C until freezing to maintain metagenome integrity
- Annotating metadata for each sample in a coherent and standardized manner allows data analysis and interpretation that would not be possible otherwise
- To ensure metadata standardization, participants had to use a specific submission form designed on KoboToolbox (practically demonstrated)

Ready-to-use resources

In the following pages, we provide:

- Bilingual (ITA and ENG) version of the evaluation survey administered to participants and non-participants
- Metadata submission form template
- Step-by-step guide for participatory environmental microbiome campaigns
- Illustrated sampling protocol
- Example checklists for environmental microbiome sampling

Metadata Submission Form Template

Bicocca Sampling Day 2023

- ENG
- ITA (original text)

Insert the Sample Number
Inserisci il Numero del campione
(es: 11A, 2B, 413C, ...)

Where are you sampling?
Dove stai campionando?

- [ ] Vivaio
- [ ] Piazza della Scienza
- [ ] Piazza dell'Ateneo Nuovo

What type of sample are you collecting?
Che tipo di campione stai raccogliendo?

- [ ] Superficie Surface
- [ ] Terreno Soil
- [ ] Acqua Water

What type of surface have you sampled?
Quale superficie hai campionato?
Conditional field (only if sample type = surface)

Point and Shoot! Take a picture of the sampling point
Punta e Scatta! Fai una foto al punto campionato

Click here to upload file. (< 10MB)

Record the location of the sampling point
Registra la posizione del punto campionato
Location records are taken automatically

latitude (x.y °)

longitude (x.y °)

altitude (m)

accuracy (m)

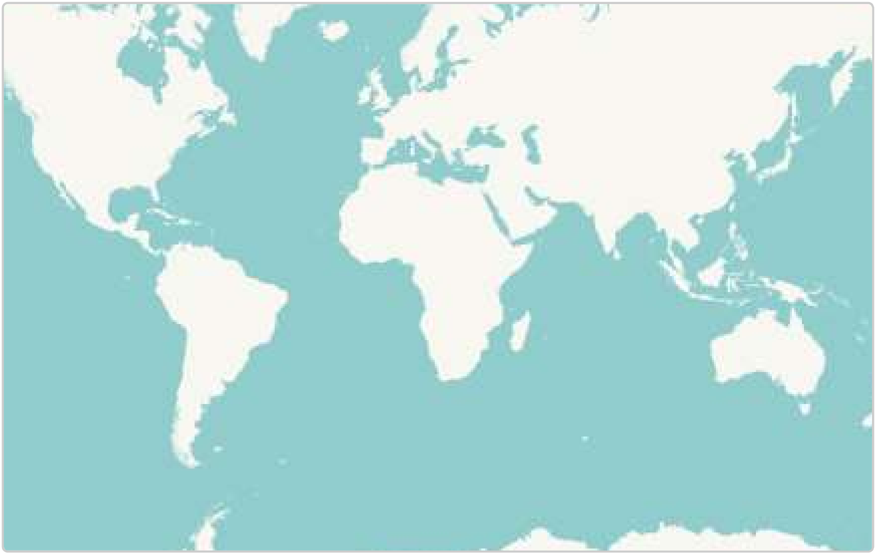

Register the sampling time

Registra l'ora del campionamento

Date and time records are takenautomatically

yyyy-mm-dd

hh:mm

Do you have more to annotate?

Altro da annotare?

Evaluation Survey – *Bicocca Sampling Days*

● **ITA (original text)**

● **ENG translation**

Indagine Progetti Scientifici Partecipati e Microbiota

Survey on Participatory Scientific Projects and Microbiota

Introduction

L'obbiettivo del seguente questionario, che é completamente **anonimo**, é di valutare l'impatto educativo dell'attivitá di Student-Science *Bicocca Sampling Day* relativamente a temi riguardanti lo studio del microbiota.
La valutazione avverrá analizzando le risposte al seguente questionario di **studenti che hanno e non hanno partecipato all'attivitá**.
Chiediamo gentilmente a chi deciderá di rispondere alle nostre domande di dedicarci il tempo e l'attenzione necessari, in quanto queste risposte forniranno importanti indicazioni per migliorare le attivitá future e per rendere il coinvolgimento degli studenti una pratica standard della ricerca scientifica.
Le domande di questo questionario riguardano il corso di studi di attinenza, e l'attitudine verso la scienza e verso lo studio del microbiota.
Tempo stimato per la compilazione: 10 minuti
Per qualsiasi esigenza, siamo contattabili a questo indirizzo: biome.research.team@gmail.com
Grazie per la collaborazione!

The objective of this questionnaire, which is completely **anonymous**, is to evaluate the educational impact of the *Student-Science Bicocca Sampling Day* activity, specifically regarding topics related to microbiota research.
The evaluation will be conducted by analyzing the responses to this questionnaire from **students who participated in the activity as well as those who did not.**
We kindly ask those who choose to answer our questions to dedicate the necessary time and attention, as the responses will provide valuable insights to improve future activities and to make student involvement a standard practice in scientific research.
The questions in this survey concern your field of study and your attitude toward science and microbiota research.
Estimated time to complete: 10 minutes.
For any inquiries, you can contact us at: biome.research.team@gmail.com
Thank you for your cooperation!

SECTION 1

1) **A quale corso di studi sei iscritto?**
   Which study program are you enrolled in?
   Seleziona una opzione
   Select one option
   a. Scienze Biologiche (laurea triennale)
      Biological Sciences (Bachelor's Degree)
   b. Biotecnologie (laurea triennale)
      Biotechnology (Bachelor's Degree)
   c. Biologia (laurea magistrale)
      Biology (Master's Degree)
   d. Biotecnologie Mediche (laurea magistrale)
      Medical Biotechnology (Master's Degree)
   e. Biotecnologie Industriali (laurea magistrale)
      Industrial Biotechnology (Master's Degree)
   f. Tecnologie Convergenti per i Sistemi Biomolecolari (Dottorato)
      Converging Technologies for Biomolecular Systems (PhD)
   g. Altro...
      Other...

2) **Quale anno frequenti?**
   Which year are you attending?
   Seleziona una opzione
   Select one option
   a. Primo anno
      First year
   b. Secondo anno
      Second year
   c. Terzo anno (solo per lauree triennali e dottorato)
      Third year (only for Bachelor's Degrees and PhD programs)
   d. Sono fuori corso
      I am beyond the regular course duration

3) **Hai partecipato ad una o piú edizioni dell’evento “Bicocca Sampling Day”?**
   Have you participated in one or more editions of the "Bicocca Sampling Day" event?

Seleziona una opzione

Select one option

a. Sí Yes -> continue to section 2
b. No No -> continue to section 3

SECTION 2 - Bicocca Sampling Days

4) **A quale/quali edizioni del "Bicocca Sampling Day" hai partecipato?**

Which edition(s) of the "Bicocca Sampling Day" did you attend?

Seleziona una o piú opzioni

Select one or more options

a. 1
b. 2
c. 3
d. 4

5) **Attualmente** ho le competenze necessarie a...

I **currently** have the skills necessary to...

Indica quanto sei d'accordo o in disaccordo con ciascuna delle seguenti dichiarazioni selezionando una delle 5 opzioni (Fortemente in disaccordo, In disaccordo, Neutrale, D'accordo, Fortemente d'accordo). **Per favore, rispondi in base a quello che senti davvero, e non in base a come pensi si possano sentire gli altri.**

Indicate how much you agree or disagree with each of the following statements by selecting one of the 5 options (Strongly Disagree, Disagree, Neutral, Agree, Strongly Agree). **Please answer based on your true feelings, not based on what you think others might feel.**

	**Fortemente in disaccordo** **Strongly Disagree**	**In disaccordo** **Disagree**	**Neutrale** **Neutral**	**D'accordo** **Agree**	**Fortemente d'accordo** **Strongly Agree**
**Osservare/registrare dati relativi al campionamento del microbiota ambientale** **Observe/record data related to the sampling of environmental microbiota**	○	○	○	○	○

**Identificare accuratamente possibili punti di campionamento del microbiota ambientale** Accurately identify potential sampling points for environmental microbiota	○	○	○	○	○
**Capire il protocollo di campionamento del microbiota ambientale** Understand the protocol for environmental microbiota sampling	○	○	○	○	○
**Sottomettere correttamente i metadati relativi al campionamento del microbiota ambientale tramite KoboToolbox** Successfully submit metadata related to environmental microbiota sampling through KoboToolbox	○	○	○	○	○
**Collezionare dati sui campioni di microbiota ambientale in maniera standardizzata** Collect data on environmental microbiota samples in a standardized manner	○	○	○	○	○
**Progettare una campagna di campionamento di microbiota ambientale** Design an environmental microbiota sampling campaign	○	○	○	○	○
**Comunicare ad altri il protocollo di campionamento del microbiota ambientale** Communicate the environmental	○	○	○	○	○

**microbiota sampling protocol to others**					
**Istruire gli altri per partecipare all'attività** **Train others to participate in the activity**	◯	◯	◯	◯	◯

6) **Prima di partecipare ai Bicocca Sampling Days** avevo le competenze necessarie a...
Prior to participating in the Bicocca Sampling Days I had the skills necessary to...

Indica quanto sei d'accordo o in disaccordo con ciascuna delle seguenti dichiarazioni selezionando una delle 5 opzioni (Fortemente in disaccordo, In disaccordo, Neutrale, D'accordo, Fortemente d'accordo). **Per favore, rispondi in base a quello che senti davvero, e non in base a come pensi si possano sentire gli altri.**
Indicate how much you agree or disagree with each of the following statements by selecting one of the 5 options (Strongly Disagree, Disagree, Neutral, Agree, Strongly Agree). **Please answer based on your true feelings, not based on what you think others might feel.**

	**Fortemente in disaccordo** **Strongly Disagree**	**In disaccordo** **Disagree**	**Neutrale** **Neutral**	**D'accordo** **Agree**	**Fortemente d'accordo** **Strongly Agree**
**Osservare/registrare dati relativi al campionamento del microbiota ambientale** **Observe/record data related to the sampling of environmental microbiota**	◯	◯	◯	◯	◯
**Identificare accuratamente possibili punti di campionamento del microbiota ambientale** **Accurately identify potential sampling points for environmental microbiota**	◯	◯	◯	◯	◯
**Capire il protocollo di campionamento del microbiota ambientale** **Understand the protocol for**	◯	◯	◯	◯	◯

**environmental microbiota sampling**					
**Sottomettere correttamente i metadati relativi al campionamento del microbiota ambientale tramite KoboToolbox** **Successfully submit metadata related to environmental microbiota sampling through KoboToolbox**	○	○	○	○	○
**Collezionare dati sui campioni di microbiota ambientale in maniera standardizzata** **Collect data on environmental microbiota samples in a standardized manner**	○	○	○	○	○
**Progettare una campagna di campionamento di microbiota ambientale** **Design an environmental microbiota sampling campaign**	○	○	○	○	○
**Comunicare ad altri il protocollo di campionamento del microbiota ambientale** **Communicate the environmental microbiota sampling protocol to others**	○	○	○	○	○
**Istruire gli altri per partecipare all'attività** **Train others to participate in the activity**	○	○	○	○	○

SECTION 3 – Attitudes Towards Science

7) Queste dichiarazioni si riferiscono a come ti senti nei confronti della **scienza in generale**
   These statements refer to how you feel about **science in general**
   Indica quanto sei d'accordo o in disaccordo con ciascuna delle seguenti dichiarazioni selezionando una delle 5 opzioni (Fortemente in disaccordo, In disaccordo, Neutrale, D'accordo,

Fortemente d'accordo). **Per favore, rispondi in base a quello che senti davvero, e non in base a come pensi si possano sentire gli altri.**
Indicate how much you agree or disagree with each of the following statements by selecting one of the 5 options (Strongly Disagree, Disagree, Neutral, Agree, Strongly Agree). **Please answer based on your true feelings, not based on what you think others might feel.**

	**Fortemente in disaccordo** Strongly Disagree	**In disaccordo** Disagree	**Neutrale** Neutral	**D'accordo** Agree	**Fortemente d'accordo** Strongly Agree
**La scienza e la tecnologia stanno rendendo le nostre vite piú salutari, piú facili e piú comode** Science and technology are making our lives healthier, easier, and more comfortable	○	○	○	○	○
**I benefici della scienza sono maggiori di qualsiasi effetto dannoso** The benefits of science are greater than any harmful effects	○	○	○	○	○
**La scienza rende il nostro stile di vita troppo veloce** Science makes our way of life move too fast	○	○	○	○	○
**Dipendiamo troppo dalla scienza e non abbastanza dalla fede** We depend too much of science and not enough on faith	○	○	○	○	○

8) Queste dichiarazioni si riferiscono a come ti senti nei confronti del **rapporto uomo-natura**
These statements refer to how you feel about the **relationship between humans and nature**

Indica quanto sei d'accordo o in disaccordo con ciascuna delle seguenti dichiarazioni selezionando una delle 5 opzioni (Fortemente in disaccordo, In disaccordo, Neutrale, D'accordo,

Fortemente d'accordo). **Per favore, rispondi in base a quello che senti davvero, e non in base a come pensi si possano sentire gli altri.**
Indicate how much you agree or disagree with each of the following statements by selecting one of the 5 options (Strongly Disagree, Disagree, Neutral, Agree, Strongly Agree). **Please answer based on your true feelings, not based on what you think others might feel.**

	Fortemente in disaccordo Strongly Disagree	In disaccordo Disagree	Neutrale Neutral	D'accordo Agree	Fortemente d'accordo Strongly Agree
L'uomo é stato creato per governare sul resto della natura Humans were created to rule over the rest of nature	○	○	○	○	○
Le persone hanno il diritto di modificare l'ambiente naturale in base alle loro esigenze People have the right to modify the natural environment to suit their needs	○	○	○	○	○
Le piante e gli animali esistono principalmente per essere usati dalle persone Plants and animals exist primarily to be used by people	○	○	○	○	○
Le persone non hanno bisogno di adattarsi all'ambiente naturale perché possono modificarlo per soddisfare le loro esigenze People need not to adapt to the natural environment because they can remake it to suit their needs	○	○	○	○	○

SECTION 4 - Attitude toward the study of environmental microbiota

9) **Quando senti o leggi i termini "campionamento del microbiota ambientale" hai:**
When you hear or read the terms "environmental microbiota sampling," do you feel:
Seleziona una opzione
Select one option
a. una chiara comprensione di che cosa significa
a clear understanding of what it means
b. un senso generale di che cosa significa
a general sense of what it means
c. poca comprensione di che cosa significa
little understanding of what it means

10) Se hai risposto a. (una chiara comprensione di che cosa significa) oppure b. (un senso generale di che cosa significa) nella domanda precedente, raccontaci con parole tue cosa significa campionare il microbiota ambientale e come avviene questo campionamento
If you answered a. (a clear understanding of what it means) or b. (a general sense of what it means) in the previous question, please tell us in your own words what it means to sample environmental microbiota and how this sampling is carried out.

SECTION 5 - Attitude toward the study of environmental microbiota

11) **Indica se le seguenti affermazioni sul campionamento del microbiota ambientale sono "Vere" o "False":**
Indicate whether the following statements about environmental microbiota sampling are "True" or "False":

	Vero<br>True	Falso<br>False
**L'utilizzo di tamponi sterili NON é efficace per il campionamento del microbiota ambientale**<br>**The use of sterile swabs is NOT effective for environmental microbiota sampling**	○	○
**Il campionamento del microbiota prevede la compilazione dei metadati**<br>**The sampling of microbiota involves the compilation of metadata**	○	○
**I campioni raccolti vanno conservati a temperatura ambiente**<br>**The collected samples should be stored at room temperature**	○	○
**Raccogliere piú repliche per ogni punto campionato é una buona pratica**<br>**Collecting multiple replicates from each sampling point is a good practice**	○	○
**Annotare il tipo di superficie campionata non é rilevante perché le comunitá microbiche ambientali non sono influenzate dal tipo di superficie**<br>**Noting the type of surface sampled is not relevant because environmental microbial communities are not influenced by the type of surface**	○	○
**La biodiversitá microbica nei contesti urbani é maggiore che nei contesti rurali**<br>**Microbial biodiversity in urban contexts is greater than in rural contexts**	○	○

**Senza batteri simbionti NON potremmo vivere**<br>**Without symbiotic bacteria, we could not live**	○	○
**I microrganismi sono ubiquitari**<br>**Microorganisms are ubiquitous**	○	○
**I microrganismi influenzano solo negativamente la nostra salute**<br>**Microorganisms only affect our health negatively**	○	○
**Studiare i campioni di microbioma ambientale tramite crescita in piastra dei microorganismi é l'approccio piú informativo**<br>**Studying environmental microbiome samples through microorganism growth on plates is the most informative approach**	○	○

12) Queste dichiarazioni si riferiscono a come ti senti nei confronti dell'apprendimento e della **comprensione di argomenti scientifici specifici (microbiota)**
These statements refer to how you feel about learning and **understanding specific scientific topics (microbiota)**

Indica quanto sei d'accordo o in disaccordo con ciascuna delle seguenti dichiarazioni selezionando una delle 5 opzioni (Fortemente in disaccordo, In disaccordo, Neutrale, D'accordo, Fortemente d'accordo). **Per favore, rispondi in base a quello che senti davvero, e non in base a come pensi si possano sentire gli altri.**
Indicate how much you agree or disagree with each of the following statements by selecting one of the 5 options (Strongly Disagree, Disagree, Neutral, Agree, Strongly Agree). **Please answer based on your true feelings, not based on what you think others might feel.**

	**Fortemente in disaccordo**<br>**Strongly Disagree**	**In disaccordo**<br>**Disagree**	**Neutrale**<br>**Neutral**	**D'accordo**<br>**Agree**	**Fortemente d'accordo**<br>**Strongly Agree**
**Penso di poter capire bene argomenti relativi al microbiota**<br>**I think I'm pretty good at understanding topics about the microbiota**	○	○	○	○	○
**Rispetto ai miei coetanei, penso di capire velocemente nuovi concetti relativi al microbiota**<br>**Compared to other people my**	○	○	○	○	○

age, I think I can quickly understand new topics about the microbiota					
Penso di impiegare molto tempo a capire nuovi concetti relativi al microbiota<br>It takes me a long time to understand new topics about the microbiota	○	○	○	○	○
Ho fiducia nella mia capacità di spiegare concetti relativi al microbiota ad altri<br>I feel confident in my ability to explain concepts about the microbiota to others	○	○	○	○	○

13) Queste dichiarazioni si riferiscono a come ti senti nei confronti dello **svolgimento di attivitá scientifiche specifiche (campionamento del microbiota)**
These statements refer to how you feel about **carrying out specific scientific activities (environmental microbiota sampling)**

Indica quanto sei d'accordo o in disaccordo con ciascuna delle seguenti dichiarazioni selezionando una delle 5 opzioni (Fortemente in disaccordo, In disaccordo, Neutrale, D'accordo, Fortemente d'accordo). **Per favore, rispondi in base a quello che senti davvero, e non in base a come pensi si possano sentire gli altri.**
Indicate how much you agree or disagree with each of the following statements by selecting one of the 5 options (Strongly Disagree, Disagree, Neutral, Agree, Strongly Agree). **Please answer based on your true feelings, not based on what you think others might feel.**

	Fortemente in disaccordo<br>Strongly Disagree	In disaccordo<br>Disagree	Neutrale<br>Neutral	D'accordo<br>Agree	Fortemente d'accordo<br>Strongly Agree

**Penso di poter seguire bene le istruzioni relative al campionamento del microbiota ambientale** I think I'm pretty good at following instructions for environmental microbiota sampling	○	○	○	○	○
**Rispetto ai miei coetanei, penso di saper campionare abbastanza bene il microbiota ambientale** Compared to other people my age, I think I can sample the environmental microbiota pretty well	○	○	○	○	○
**Penso di impiegare molto tempo a capire come campionare il microbiota ambientale** It takes me a long time to understand how to sample the environmental microbiota	○	○	○	○	○
**Ho fiducia nella mia capacità di spiegare ad altri come si effettua il campionamento del microbiota ambientale** I feel confident about my ability to explain how to sample the environmental microbiota to others	○	○	○	○	○

HOW TO

DESIGN A PARTICIPATORY ENVIRONMENTAL MICROBIOME SAMPLING CAMPAIGN

A STEP-BY-STEP GUIDE BASED ON THE BICOCCA SAMPLING DAYS MODEL

Project Design

START

Do you have a clear and defined RESEARCH QUESTION?

YES / NO

Do you have all this information?

SAMPLING AREA
- What are your environmental area of interest? — How many? —

TIMEPOINTS
- What are your timepoints of interest? — How many? —

NO / YES

For each Sampling Area:

SAMPLE TYPES
- What Sample Types will participants collect?
- How many Sampling Points for each Sample Type?

How many? — Total: —

REPLICATES
- How many replicates do you want to collect for each Sampling Point? — How many? —

Multiply all numbers to obtain the **Total Sample Number**

Have you identified all the Sampling Points you are interested in?

NO / YES

List out your Sampling Points and group them according to the Sampling Area to obtain the **Sample Checklists**

Make sure you have all required **Permissions** to access and sample the areas of interest

Have you identified your Target PARTICIPANTS?

YES / NO

According to your Target Participants, you should organise a tailored

RECRUITMENT CAMPAIGN

Give your Project an Identity with a **TITLE**, a **LOGO**, and a reference **Social Media** page

Social media are effective in reaching young adults. To reach other target populations you should also consider flyers, mailing lists, local communicative interventions, …

INSTITUTIONS' INVOLVEMENT
- Are there local institutions that could support your project and help with participant recruitment?

SPONSORSHIP
- Are there organizations or societies that could promote your project?

ex: International Microorganism Day

NO

Do you have a clear communication/recruitment plan?

YES

Prepare a **Rergistration Form** to allow participants to register for the activity and to provide all the related details.

Other useful papers to prepare:
- **Participants Register** to note participants' presence, certificate of attendance requests, and disclosure form agreements.
- **Disclosure form** on the use of images of the subject appearing in photographs and videos
- **Certificate of Attendance** to be delivered to participants

Material Preparation

If you know when, where and what your participants will sample and you know how many participants will join your event, **it's time to get started with the material preparation…**

Sampling Materials

Example:

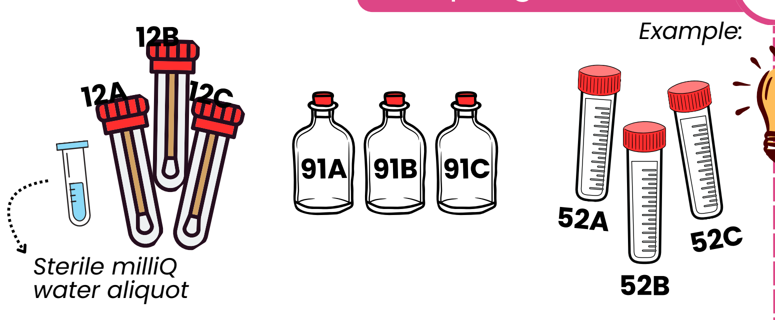

Prepare the materials according to the **Total Sample Number**

Protocols

Illustrated and detailed protocols can be printed out and provided to participants as a take-home material and as a support durng sampling activities.

Metadata Submission Form

Make sure all the metadata you need to be associated with your samples are included in the form

HERE SOME EXAMPLES OF FIELD YOU MAY CONSIDER TO INSERT IN YOUR METADATA SUBMISSION FORM

- **SAMPLE NAME/NUMBER** this **text field** allows you to associate the sample metadata with specific collected samples
- **SAMPLING AREA NAME** if your sampling campaign includes several sampling areas, consider a **drop-down menu** from which participants can choose one option
- **SAMPLE TYPE** if your sampling campaign includes different sampling types, consider a **drop-down menu** from which participants can choose one option
  - **Surface type** if you are interested in classifying your samples according to the surface type, add a **text field** to let participants specify
- **DATE AND TIME** if your campaign includes several sampling days, this field ensures the time differentiation of samples
- **PHOTO** Asking participants to photograph what they are sampling while they are sampling is a way to record numerous pieces of information that are not explicitly recordable (how the sampling has been performed, more detail on the sampling site, …). Photos are also fundamental to disambiguate possible incoherences in the metadata
- **GEOLOCALIZATION** recording the coordinates of each sample and replicate will allow samples georeferencing

Theoretical Lesson

To define the **CORE CONCEPTS** of yor lesson, keep in mind that if you want to evaluate participants gain of knowledge, your **Evaluation Plan** should be tailored to the concept of your lesson

CORE CONCEPTS
Example from the BSDs model:

- Microorganisms are **ubiquitous** and live in diverse and dynamic ecosystems
- Microorganisms, cellular and viral, can **interact with** both human and non-human **hosts** in beneficial, neutral, or detrimental ways
- Microbes are **essential** for life as we know it and the processes that support life
- **Metagenomics** is more informative than isolate cultures for microbiome characterization
- **Human impact** on the environment influences the **evolution** of microorganisms and vice versa
- Swabs, collection tubes, and bottles are the **tools** used to sample environmental microbiomes
- Sample **replicates** guarantee robust and reliable data
- Since microorganisms are ubiquitous, sampling and sample processing must be performed with caution avoiding user-derived or cross-sample **contamination**
- Collected samples must be conserved at 4°C until freezing to maintain **metagenome integrity**
- To ensure **metadata standardization**, participants had to use a specific submission form designed on KoboToolbox
- Annotating **metadata** for each sample in a coherent and standardized manner allows data analysis and interpretation that would not be possible otherwise

Have you prepared all the required MATERIALS?

NO / YES

Evaluation Plan

Evaluation Plan

What do you want to evaluate?
- Attitudes toward Science and the Environment
- Skills for Environmental Microbiome Inquiry
- Self-Efficacy for Learning and Doing Environmental Microbiome Sampling
- Understanding of the "Environmental Microbiome Sampling" process
- Knowledge of the "Environmental Microbiome Sampling" process

When will you do the evaluation?
It is essential that you define ***a priori*** the type of evaluation assessment you need: pre-post , retroactive, post-event only,…

SCALES
- **STANDARD** scales
- **CUSTOMIZED** scales
- **NOVEL** scales

! New scales need to be validated!

→ SURVEY ← after administration →

SCORING
Establish ***a priori*** the scoring for each item of the scales. For standard and customized scales, follow the scoring instructions provided by the authors.

→ ANALYSIS and RESULTS

If you have planned and prepared everything, you are **READY** to host your participatory sampling event!

Event Outline

Event Outline

1 RECEPTION • Duration: ____________

Welcome your participants registering their presence on the **Register**, collect signed **Disclosure Forms** and annotate **Certificate of Attendance** requests.

PRE-ACTIVITY SURVEY
If your evaluation plan requires **pre** and **post** surveys, administer the **pre** survey before the activities start.

2 LESSON AND TRAINING • Duration: ____________

Hand out the **Protocols**, and give access to the **Metadata Submission Form**.

Introduce and explain the **Research Question** and the **Core Concepts**.

3 SAMPLING

Provide **Sampling Materials** and divide the participants into working groups, each will be given a **Sample Checklist** as a guide and access to the **Metadata Submission Form**.

Supervise and be available during the activities.

4 FINAL CONVIVIAL MOMENT

Organize a final informal moment to conclude the event, and collect feedback.

POST-ACTIVITY SURVEY
Administer the **post** survey after the event. You can decide to administer it on-site or send a digital survey to the participants *a posteriori*.

TIPS AND TRICKS

1. Include sampling points at the participants' discretion. This allows participants to commit to the research question and reflect with criticisms. In addition their contribution to the sampling design adds value to the project via non stereotypical and field-based perspectives
2. Divide into working groups to promote social learning dynamics.
3. Organize materials and tool identifiers in replicates to facilitate replicates sampling.
4. The preferential use of multiple-choice questions rather than open-ended ones implies less manual curation but less additional participant-derived information is collected. Photographic documentation enhances metadata accuracy. KoboToolbox provides automatic localization, and date and time recordings.

Finanziato dall'Unione europea NextGenerationEU
Italiadomani PIANO NAZIONALE DI RIPRESA E RESILIENZA
MUR
MUSA
UNIVERSITÀ DEGLI STUDI DI MILANO BICOCCA 25 1998 | 2023
POLITECNICO MILANO 1863
BIOME RESEARCH Team

SAMPLING PROTOCOL

HOW TO SAMPLE THE ENVIRONMENTAL MICROBIOTA?

SURFACES

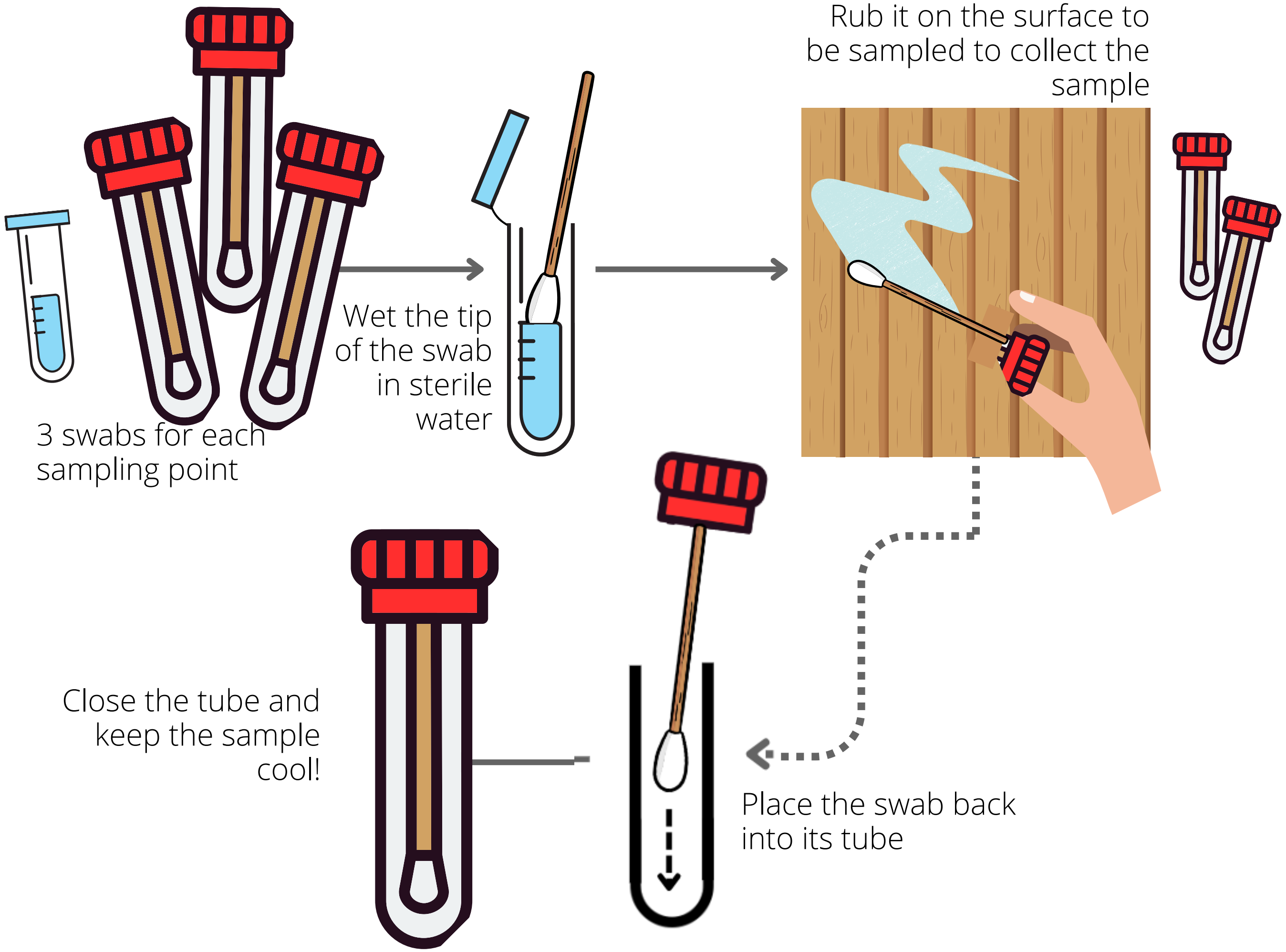

SOIL/WATER

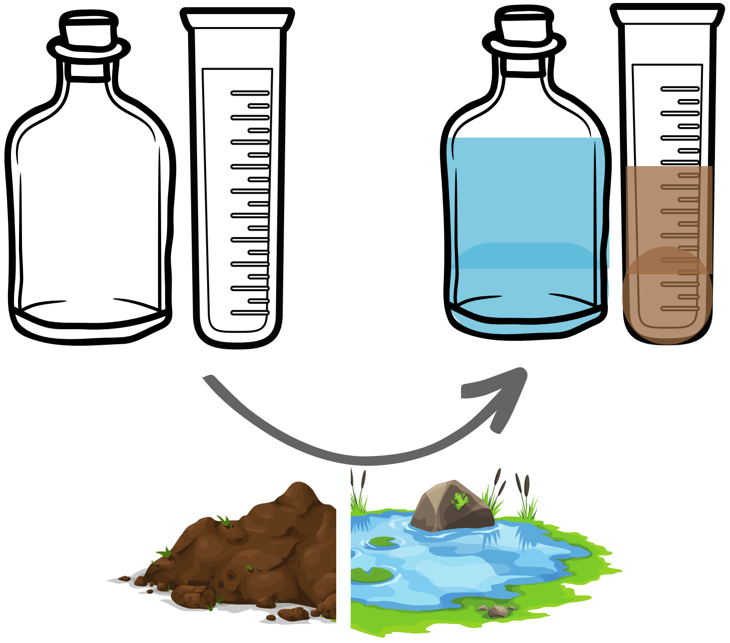

Use the container to collect a portion of soil (< 50 mL) or water (1 L)

This protocol was produced for the participatory sampling event **Bicocca Sampling Days**

Finanziato dall'Unione europea NextGenerationEU

Ministero dell'Università e della Ricerca

Italiadomani PIANO NAZIONALE DI RIPRESA E RESILIENZA

MUSA

UNIVERSITÀ DEGLI STUDI DI MILANO BICOCCA 25 1998 | 2023

BIOME RESEARCH Team

VIVAIO BICOCCA

BICOCCA SAMPLING DAYS GROUP 1

CHECK LIST VIVAIO BICOCCA

- [ ] **Water** of the pond (1 bottle) : _____
- [ ] **Soil** near the entrance (3 replicates) : _______________
- [ ] **Tree** trunk surface (3 replicates) : _______________
- [ ] **Leaves** surface of the same tree (3 replicates): _________
- [ ] **Entrance gate** surface (3 replicates) : ______________
- [ ] Surface of choice (3 replicates) : _______________
- [ ] Surface of choice (3 replicates) : _______________
- [ ] Surface of choice (3 replicates) : _______________
- [ ] Surface of choice (3 replicates) : _______________

CHECK LIST PIAZZA DELLA SCIENZA (U3-U4)

- [ ] Surface of the **floor** at the entrance of U3 (3 replicates) :_______
- [ ] **Bicycle parking** area side U4 (3 replicates): __________
- [ ] Surface of choice (3 replicates) : _______________
- [ ] Surface of choice (3 replicates) : _______________
- [ ] Surface of choice (3 replicates) : _______________
- [ ] Surface of choice (3 replicates) : _______________
- [ ] Surface of choice (3 replicates) : _______________
- [ ] **Soil** : _______________

CHECK LIST PIAZZA DELL'ATENEO NUOVO

- [ ] Surface of the square **floor** (3 replicates) :_________
- [ ] **Tree** trunk surface (3 replicates) : __________________
- [ ] **Leaves** surface of the same tree (3 replicates): ___________
- [ ] Surface of choice (3 replicates) : __________________
- [ ] Surface of choice (3 replicates) : __________________
- [ ] Surface of choice (3 replicates) : __________________
- [ ] Surface of choice (3 replicates) : __________________

BICOCCA SAMPLING DAYS GROUP 2

CHECK LIST VIVAIO

- ☐ **Water** of the pond (1 bottle) : _____
- ☐ **Soil** near the pond (3 replicates) : _______________
- ☐ Surface of the **tool shed** (3 replicates) : _____________
- ☐ Surface of the **tools** in the shed (3 replicates) : _______________
- ☐ Surface of the **trunck stool** (3 replicates) : _______________
- ☐ Surface of choice (3 replicates) : _______________
- ☐ Surface of choice (3 replicates) : _______________
- ☐ Surface of choice (3 replicates) : _______________
- ☐ Surface of choice (3 replicates) : _______________

CHECK LIST PIAZZA DELLA SCIENZA

- ☐ **Railing** near the entrance of U3 (3 repliates) : _______________
- ☐ **Traffic light button**, U3 side (3 replicates) : __________
- ☐ **Wall** near the entrance of U3 (3 replicates) : __________
- ☐ Surface of choice (3 replicates) : _______________
- ☐ Surface of choice (3 replicates) : _______________
- ☐ Surface of choice (3 replicates) : _______________
- ☐ Surface of choice (3 replicates) : _______________
- ☐ Soil : _______________

CHECK LIST PIAZZA DELL'ATENEO NUOVO

- [ ] Surface of the square **floor** (3 replicates) :________
- [ ] **Bench** surface **NOT in the smoking area** (3 replicates) : ______
- [ ] **Wall** surface near the entrance of U6 (3 replicates) : __________
- [ ] Surface of choice (3 replicates) : ________________
- [ ] Surface of choice (3 replicates) : ________________
- [ ] Surface of choice (3 replicates) : ________________
- [ ] Surface of choice (3 replicates) : ________________

BICOCCA SAMPLING DAYS GROUP 3

CHECK LIST VIVAIO

- [ ] **Water** of the pond (1 bottle) : ______
- [ ] **Soil** near the Apiary (3 replicates) : __________________
- [ ] **Wall** surface bordering the railway (3 replicates) : ___________
- [ ] **Tree** trunk surface (3 replicates) : __________________
- [ ] **Leaves** surface of the same tree (3 replicates): ___________
- [ ] Surface of choice (3 replicates) : __________________
- [ ] Surface of choice (3 replicates) : __________________
- [ ] Surface of choice (3 replicates) : __________________
- [ ] Surface of choice (3 replicates) : __________________

CHECK LIST PIAZZA DELLA SCIENZA

- [ ] **Floor** near the entrance of U4 (3 replicates) : _____________
- [ ] **Wall** near the entrance of U4 (3 replicates) : ___________
- [ ] **Intercom buttons** near the entrance of U4 (3 replicates):_______
- [ ] Surface of choice (3 replicates) : __________________
- [ ] Surface of choice (3 replicates) : __________________
- [ ] Surface of choice (3 replicates) : __________________
- [ ] Surface of choice (3 replicates) : __________________
- [ ] Soil: __________________

CHECK LIST PIAZZA DELL'ATENEO NUOVO

- [ ] **Bench** surface in the **smoking area** (3 replicates) : ______
- [ ] Tree trunk surface (3 replicates) : __________________
- [ ] Leaves surface of the same tree (3 replicates): ___________
- [ ] Surface of choice (3 replicates) : __________________
- [ ] Surface of choice (3 replicates) : __________________
- [ ] Surface of choice (3 replicates) : __________________
- [ ] Surface of choice (3 replicates) : __________________

BICOCCA SAMPLING DAYS   GROUP 4

CHECK LIST VIVAIO

- [ ] **Water** of the pond (1 bottle) : _____
- [ ] **Soil** near the boundary with the railway (3 replicates) : _______
- [ ] Back **wall** surface (3 replicates) : _________
- [ ] Surface of the **tool shed** (3 replicates) : _____________
- [ ] Surface of the **tools** in the shed (3 replicates) : _______________
- [ ] Surface of choice (3 replicates) : ________________
- [ ] Surface of choice (3 replicates) : ________________
- [ ] Surface of choice (3 replicates) : ________________
- [ ] Surface of choice (3 replicates) : ________________

CHECK LIST PIAZZA DELLA SCIENZA

- [ ] **Railing** near the entrance of U4 (3 replicates) : ________________
- [ ] Entrance **door** of U4 (3 replicates) : __________
- [ ] **Intercom buttons** near the entrance of U3 (3 replicates) : _____
- [ ] Surface of choice (3 replicates) : ________________
- [ ] Surface of choice (3 replicates) : ________________
- [ ] Surface of choice (3 replicates) : ________________
- [ ] Surface of choice (3 replicates) : ________________
- [ ] Soil : ________________

CHECK LIST PIAZZA DELL'ATENEO NUOVO

- [ ] **Floor** surface at the entrance of U6 (3 replicates) :________
- [ ] **Glass** surface of the U6 building (3 replicates) :________
- [ ] Surface of choice (3 replicates) : _________________
- [ ] Surface of choice (3 replicates) : _________________
- [ ] Surface of choice (3 replicates) : _________________
- [ ] Surface of choice (3 replicates) : _________________

BICOCCA SAMPLING DAYS   GROUP 5

CHECK LIST VIVAIO

- ☐ **Water** of the pond (1 bottle) : ______
- ☐ **Terreno** in fondo al vivaio, vicino al confine (3 repliche) : _____
- ☐ Surface of the **trunck stool** (3 replicates) : __________________
- ☐ **Tree** trunk surface (3 replicates) : __________________
- ☐ **Leaves** surface of the same tree (3 replicates): ___________
- ☐ Surface of choice (3 replicates) : __________________
- ☐ Surface of choice (3 replicates) : __________________
- ☐ Surface of choice (3 replicates) : __________________
- ☐ Surface of choice (3 replicates) : __________________

CHECK LIST PIAZZA DELLA SCIENZA

- ☐ Entrance **door** of U3 (3 replicates) : __________________
- ☐ **Wall** (3 replicates) : ___________
- ☐ Surface of choice (3 replicates) : __________________
- ☐ Surface of choice (3 replicates) : __________________
- ☐ Surface of choice (3 replicates) : __________________
- ☐ Surface of choice (3 replicates) : __________________
- ☐ Surface of choice (3 replicates) : __________________
- ☐ Soil : __________________

CHECK LIST PIAZZA DELL'ATENEO NUOVO

- [ ] **Floor** surface of the square (3 replicates) :________
- [ ] **Bench** surface **NOT in the smoking area** (3 replicates) : ______
- [ ] **Wall** surface (3 repliche) : __________
- [ ] Surface of choice (3 replicates) : _________________
- [ ] Surface of choice (3 replicates) : _________________
- [ ] Surface of choice (3 replicates) : _________________
- [ ] Surface of choice (3 replicates) : _________________

BICOCCA SAMPLING DAYS   GROUP 6

CHECK LIST VIVAIO

- ☐ **Water** of the pond (1 bottle) : ______
- ☐ **Terreno** al centro del vivaio (3 repliche) : ________
- ☐ Surface of the **trunck stool** (3 replicates) : __________________
- ☐ Surface of the **tool shed** (3 replicates) : _______________
- ☐ Surface of the **tools** in the shed (3 replicates) : _________________
- ☐ Surface of choice (3 replicates) : __________________
- ☐ Surface of choice (3 replicates) : __________________
- ☐ Surface of choice (3 replicates) : __________________
- ☐ Surface of choice (3 replicates) : __________________

CHECK LIST PIAZZA DELLA SCIENZA

- ☐ **Bicycle parking** on the U4 side (3 replicates) : ___________
- ☐ **Traffic light button**, U4 side (3 replicates) : ___________
- ☐ Surface of choice (3 replicates) : __________________
- ☐ Surface of choice (3 replicates) : __________________
- ☐ Surface of choice (3 replicates) : __________________
- ☐ Surface of choice (3 replicates) : __________________
- ☐ Soil : __________________

CHECK LIST PIAZZA DELL'ATENEO NUOVO

- [ ] **Floor** surface of the square (3 replicates) :________
- [ ] **Tree** trunk surface (3 replicates) : ________________
- [ ] **Leaves** surface of the same tree (3 replicates): __________
- [ ] Surface of choice (3 replicates) : ________________
- [ ] Surface of choice (3 replicates) : ________________
- [ ] Surface of choice (3 replicates) : ________________
- [ ] Surface of choice (3 replicates) : ________________

BICOCCA SAMPLING DAYS GROUP 7

CHECK LIST VIVAIO

- [ ] **Water** of the pond (1 bottle) : ______
- [ ] **Terreno** nel querceto (3 repliche) : ________
- [ ] Surface of the biodiversity **wall** (3 replicates) : ___________
- [ ] **Tree** trunk surface (3 replicates) : __________________
- [ ] **Leaves** surface of the same tree (3 replicates): ___________
- [ ] Surface of choice (3 replicates) : __________________
- [ ] Surface of choice (3 replicates) : __________________
- [ ] Surface of choice (3 replicates) : __________________
- [ ] Surface of choice (3 replicates) : __________________

CHECK LIST PIAZZA DELLA SCIENZA

- [ ] **Floor** (3 replicates) : __________________
- [ ] **Wall** (3 replicates) : ___________
- [ ] Surface of choice (3 replicates) : __________________
- [ ] Surface of choice (3 replicates) : __________________
- [ ] Surface of choice (3 replicates) : __________________
- [ ] Surface of choice (3 replicates) : __________________
- [ ] Surface of choice (3 replicates) : __________________
- [ ] Soil : __________________

CHECK LIST PIAZZA DELL'ATENEO NUOVO

- [ ] **Bench** surface in the **smoking area** (3 replicates) : ______
- [ ] **Tree** trunk surface (3 replicates) : ___________________
- [ ] **Leaves** surface of the same tree (3 replicates): ___________
- [ ] Surface of choice (3 replicates) : ___________________
- [ ] Surface of choice (3 replicates) : ___________________
- [ ] Surface of choice (3 replicates) : ___________________
- [ ] Surface of choice (3 replicates) : ___________________

BICOCCA SAMPLING DAYS GROUP 8

CHECK LIST VIVAIO

- ☐ **Water** of the pond (1 bottle) : _____
- ☐ **Soil** of the outdoor classroom (3 replicates) : _______
- ☐ Surface of the **trunck stool** (3 replicates) : ________________
- ☐ **Tree** trunk surface (3 replicates) : ________________
- ☐ **Leaves** surface of the same tree (3 replicates): __________
- ☐ Surface of choice (3 replicates) : ________________
- ☐ Surface of choice (3 replicates) : ________________
- ☐ Surface of choice (3 replicates) : ________________
- ☐ Surface of choice (3 replicates) : ________________

CHECK LIST PIAZZA DELLA SCIENZA

- ☐ **Floor** (3 replicheates) : ________________
- ☐ **Wall** (3 replicates) : __________
- ☐ Surface of choice (3 replicates) : ________________
- ☐ Surface of choice (3 replicates) : ________________
- ☐ Surface of choice (3 replicates) : ________________
- ☐ Surface of choice (3 replicates) : ________________
- ☐ Surface of choice (3 replicates) : ________________
- ☐ Soil : ________________

CHECK LIST PIAZZA DELL'ATENEO NUOVO

- [ ] **Bench** surface **NOT in the smoking area** (3 repliche) : ______
- [ ] **Tree** trunk surface (3 replicates) : ___________________
- [ ] **Leaves** surface of the same tree (3 replicates): ___________
- [ ] Surface of choice (3 replicates) : ___________________
- [ ] Surface of choice (3 replicates) : ___________________
- [ ] Surface of choice (3 replicates) : ___________________
- [ ] Surface of choice (3 replicates) : ___________________

BICOCCA SAMPLING DAYS GROUP 9

CHECK LIST VIVAIO

- ☐ **Water** of the pond (1 bottle) : _____
- ☐ **Soil** in the center of the Vivaio (3 replicates) : _______
- ☐ Surface of the **trunck stool** (3 replicates) : ________________
- ☐ **Tree** trunk surface (3 replicates) : ________________
- ☐ **Leaves** surface of the same tree (3 replicates): __________
- ☐ Surface of choice (3 replicates) : ________________
- ☐ Surface of choice (3 replicates) : ________________
- ☐ Surface of choice (3 replicates) : ________________
- ☐ Surface of choice (3 replicates) : ________________

CHECK LIST PIAZZA DELLA SCIENZA

- ☐ **Floor** (3 replicates) : ________________
- ☐ **Wall** (3 replicates) : __________
- ☐ Surface of choice (3 replicates) : ________________
- ☐ Surface of choice (3 replicates) : ________________
- ☐ Surface of choice (3 replicates) : ________________
- ☐ Surface of choice (3 replicates) : ________________
- ☐ Soil : ________________

CHECK LIST PIAZZA DELL'ATENEO NUOVO

- [ ] **Wall** surface (3 replicates) : ______
- [ ] **Tree** trunk surface (3 replicates) : __________________
- [ ] **Leaves** surface of the same tree (3 replicates): ___________
- [ ] Surface of choice (3 replicates) : __________________
- [ ] Surface of choice (3 replicates) : __________________
- [ ] Surface of choice (3 replicates) : __________________
- [ ] Surface of choice (3 replicates) : __________________

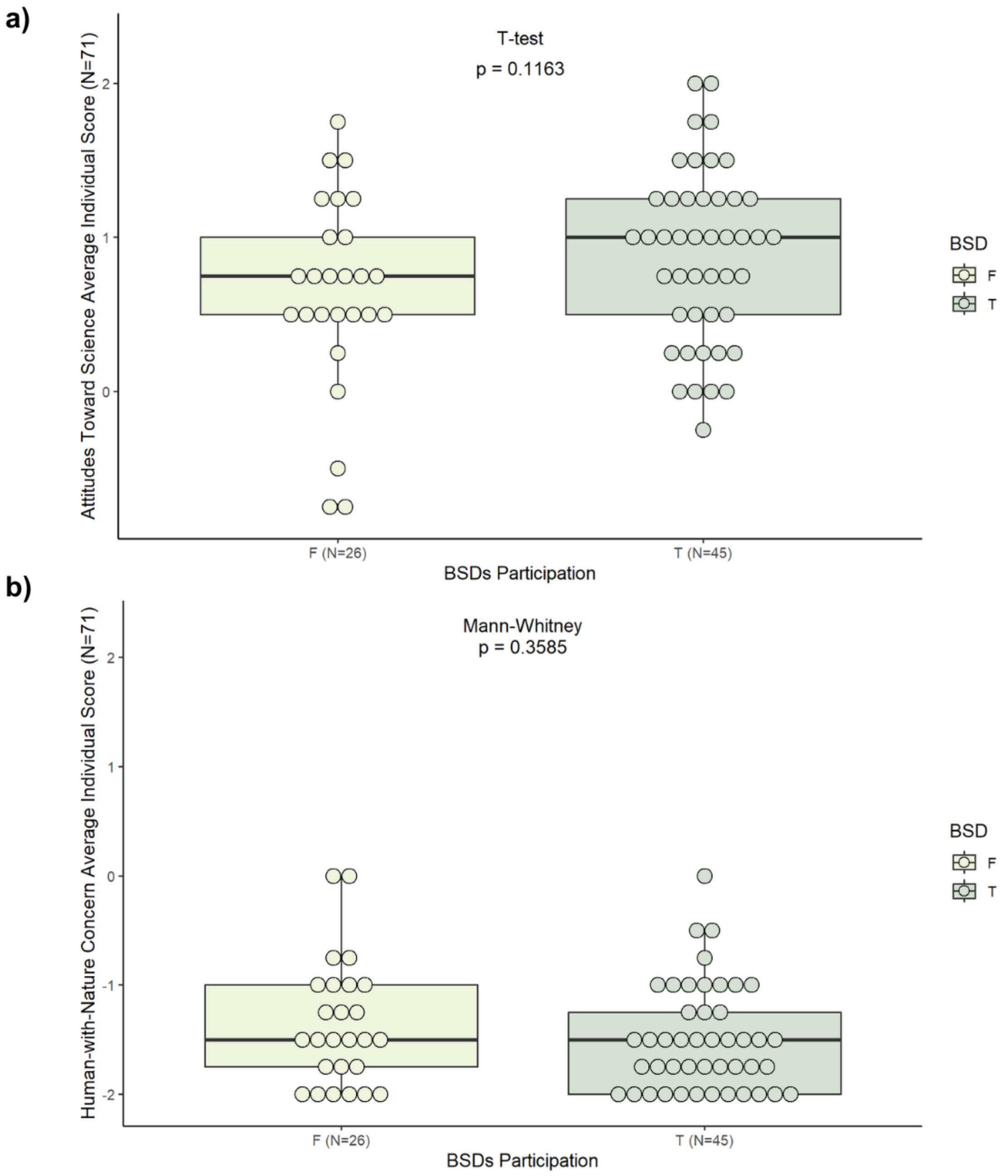

Fig. S1. Average individual scores of participants and non-participants for the MATOSS (a) and NEP (b) subscales. Statistical tests and p-values are indicated in each panel.

Table S1. Sample numbers by sampling area and generic sampling point category.
The green gradient represents the sample abundances (white = 0, green = max value).

Sampling Point	Sampling Area	BSD1	BSD2	BSD3	BSD4	tot area	tot
**Animal-related**	piazza-ateneo	0	0	0	0	0	**24**
	piazza-scienza	0	0	0	0	0	
	vivaio-bicocca	6	6	6	6	24	
**Ashtray**	piazza-ateneo	3	7	0	3	13	**16**
	piazza-scienza	3	0	0	0	3	
	vivaio-bicocca	0	0	0	0	0	
**Banister**	piazza-ateneo	0	3	6	3	12	**56**
	piazza-scienza	15	11	12	6	44	
	vivaio-bicocca	0	0	0	0	0	
**Bench**	piazza-ateneo	21	30	30	20	101	**245**
	piazza-scienza	21	6	12	15	54	
	vivaio-bicocca	27	18	24	21	90	
**Bike Parking**	piazza-ateneo	3	6	6	3	18	**54**
	piazza-scienza	15	6	6	9	36	
	vivaio-bicocca	0	0	0	0	0	
**Button**	piazza-ateneo	0	0	0	0	0	**57**
	piazza-scienza	9	18	18	12	57	
	vivaio-bicocca	0	0	0	0	0	
**Door**	piazza-ateneo	0	0	0	0	0	**24**
	piazza-scienza	6	3	6	9	24	
	vivaio-bicocca	0	0	0	0	0	
**Doorhandle**	piazza-ateneo	0	1	0	0	1	**26**
	piazza-scienza	3	10	3	9	25	
	vivaio-bicocca	0	0	0	0	0	
**Electric Tool**	piazza-ateneo	6	6	9	9	30	**33**
	piazza-scienza	0	0	0	0	0	
	vivaio-bicocca	0	0	0	3	3	
**Emergency Tool**	piazza-ateneo	6	3	0	3	12	**24**
	piazza-scienza	0	0	6	6	12	
	vivaio-bicocca	0	0	0	0	0	
**Entrance Totem**	piazza-ateneo	0	0	0	0	0	**15**
	piazza-scienza	0	0	9	6	15	
	vivaio-bicocca	0	0	0	0	0	
**Fence**	piazza-ateneo	0	0	0	0	0	**66**
	piazza-scienza	0	0	9	6	15	
	vivaio-bicocca	12	12	18	9	51	
**Floor**	piazza-ateneo	23	30	24	19	96	**218**
	piazza-scienza	18	35	24	39	116	
	vivaio-bicocca	0	3	3	0	6	
**Indoor**	piazza-ateneo	3	14	3	3	23	**38**
	piazza-scienza	0	15	0	0	15	
	vivaio-bicocca	0	0	0	0	0	
**Mushrooms**	piazza-ateneo	0	0	0	0	0	**18**
	piazza-scienza	0	0	0	0	0	
	vivaio-bicocca	0	9	6	3	18	
**Other**	piazza-ateneo	0	1	27	27	55	**64**
	piazza-scienza	0	3	0	0	3	
	vivaio-bicocca	3	3	0	0	6	
**Sign**	piazza-ateneo	15	1	10	15	41	**138**
	piazza-scienza	6	6	9	9	30	
	vivaio-bicocca	9	30	18	10	67	
**Soil**	piazza-ateneo	6	0	0	0	6	**223**
	piazza-scienza	27	27	27	27	108	
	vivaio-bicocca	30	24	27	28	109	
**Stone**	piazza-ateneo	0	0	0	0	0	**36**
	piazza-scienza	0	0	0	0	0	
	vivaio-bicocca	9	12	12	3	36	
**Table**	piazza-ateneo	0	0	0	0	0	**14**
	piazza-scienza	0	2	3	3	8	
	vivaio-bicocca	3	0	0	3	6	
**Tool**	piazza-ateneo	0	0	0	0	0	**110**
	piazza-scienza	0	0	0	0	0	
	vivaio-bicocca	18	27	33	32	110	
**Tram Rails**	piazza-ateneo	0	0	0	0	0	**12**
	piazza-scienza	3	3	3	3	12	
	vivaio-bicocca	0	0	0	0	0	
**Trash Bin**	piazza-ateneo	12	6	6	3	27	**78**
	piazza-scienza	9	21	9	12	51	
	vivaio-bicocca	0	0	0	0	0	
**Vase**	piazza-ateneo	0	0	0	0	0	**19**
	piazza-scienza	3	0	0	0	3	
	vivaio-bicocca	9	0	6	1	16	
**Vegetation**	piazza-ateneo	48	51	42	36	177	**499**
	piazza-scienza	42	9	6	6	63	
	vivaio-bicocca	75	60	48	76	259	
**Wall**	piazza-ateneo	12	12	11	18	53	**167**
	piazza-scienza	12	15	24	27	78	
	vivaio-bicocca	6	6	12	12	36	
**Water**	piazza-ateneo	0	0	0	0	0	**36**
	piazza-scienza	0	0	0	0	0	
	vivaio-bicocca	9	9	9	9	36	
**Water-related**	piazza-ateneo	3	3	6	6	18	**57**
	piazza-scienza	9	0	0	3	12	
	vivaio-bicocca	9	6	3	9	27	
**Window**	piazza-ateneo	9	12	6	12	39	**62**
	piazza-scienza	9	5	6	3	23	
	vivaio-bicocca	0	0	0	0	0	

Table S2. Summary of the submissions to the Skills for Environmental Microbiome Inquiry scale.
Items and their scoring are shown for both the current and retrospective versions of the scale. Mean scores range from 1 (poor skills perception) to 5 (high skills perception).

Skills for Environmental Microbiome Inquiry Scale - Submissions Summary

		Current **version** “I currently have the skills necessary to...”					***Retrospective*** **version** “Prior to participating in the BSDs I had the skills necessary to...”				
		Strongly Agree **5**	Agree **4**	Neutral **3**	Disagree **2**	Strongly Disagree **1**	Strongly Agree **5**	Agree **4**	Neutral **3**	Disagree **2**	Strongly Disagree **1**
**Item 1**	*...observe/record data relative to the environmental microbiome sampling.*	33%	44%	9%	9%	4%	2%	13%	31%	20%	33%
**Item 2**	*...accurately identify potential sampling point for the environmental microbiome sampling.*	27%	58%	13%	-	2%	24%	16%	16%	44%	-
**Item 3**	*...understand the environmental microbiome sampling protocol.*	47%	42%	4%	7%	-	7%	20%	27%	24%	22%
**Item 4**	*...successfully submit environmental microbiome samples metadata via the KoboToolbox platform.*	16%	20%	9%	13%	42%	4%	4%	13%	24%	53%
**Item 5**	*...collect data about environmental microbiome samples in a standardized manner.*	31%	40%	13%	31%	9%	2%	4%	16%	42%	36%
**Item 6**	*...design an environmental microbiome sampling campaign.*	9%	36%	36%	11%	9%	-	2%	9%	24%	64%
**Item 7**	*...communicate the protocol for environmental microbiome sampling to others.*	29%	51%	16%	4%	-	2%	2%	18%	27%	51%
**Item 8**	*...train others to participate in the activity.*	36%	44%	7%	4%	-	2%	7%	11%	24%	56%
		**Mean Scale Score = 3.9 (standard deviation = 0.72)**					**Mean Scale Score = 2 (standard deviation = 0.84)**				

Table S3. Summary of the submissions to the Self-Efficacy for Learning and Doing Environmental Microbiome Sampling scale.

Items and their scoring are shown for the two subscales (learning and doing) and for the participants and non-participants groups. Mean scores range from 1 (no confidence) to 5 (strong confidence). *Scoring for Item 3 and Item 7 has been reversed (Strongly Agree = 1, Agree = 2, Neutral = 3, Disagree = 4, Strongly Disagree = 5) (4).

Self-Efficacy for Learning and Doing Microbiome-related Tasks - Submissions Summary

Indicate how much you agree or disagree with each of the following statements:

		Participants					NON-Participants				
		Strongly Agree **5**	Agree **4**	Neutral **3**	Disagree **2**	Strongly Disagree **1**	Strongly Agree **5**	Agree **4**	Neutral **3**	Disagree **2**	Strongly Disagree **1**
	**LEARNING**										
**Item 1**	*I think I'm pretty good at understanding topics about the microbiota.*	13%	67%	11%	9%	-	23%	42%	31%	0%	4%
**Item 2**	*Compared to other people my age, I think I can quickly understand new topics about the microbiota.*	24%	49%	27%	-	-	15%	50%	31%	0%	4%
**Item 3***	*It takes me a long time to understand new topics about the microbiota.*	-	13%	69%	7%	11%	4%	-	27%	54%	15%
**Item 4**	*I feel confident in my ability to explain microbiota-related topics to others.*	7%	60%	27%	4%	2%	15%	31%	38%	8%	8%
		**Mean Scale Score = 3.83 (standard deviation = 0.58)**					**Mean Scale Score = 3.67 (standard deviation = 0.68)**				
	**DOING**										
**Item 5**	*I think I'm pretty good at following instructions for environmental microbiome sampling.*	38%	58%	4%	-	-	23%	65%	8%	0%	4%
**Item 6**	*Compared to other people my age, I think I can sample the environmental microbiome pretty well.*	22%	60%	18%	-	-	12%	35%	46%	4%	4%
**Item 7***	*It takes me a long time to understand how to sample the environmental microbiome.*	-	4%	11%	51%	33%	4%	12%	23%	50%	12%
**Item 8**	*I feel confident in my ability to explain how to sample the environmental microbiome to others.*	18%	67%	13%	2%	-	12%	42%	38%	4%	4%
		**Mean Scale Score = 4.12 (standard deviation = 0.44)**					**Mean Scale Score = 3.64 (standard deviation = 0.63)**				

Table S4. Summary of the submissions to the *Knowledge* and *Understanding of the "Environmental Microbiome Sampling" process* scales.

The understanding of the process was evaluated by identifying 8 pre-defined elements in the respondents' definition of "environmental microbiome sampling". All elements contribute equally to the total score (1 point each, total maximum individual score = 8). The percentage of respondents that included each element in their definition is shown. Significant differences are marked with a white-to-green gradient (white 0%, green 100%) and the relative elements are in grey. The responses are divided into students with a clear understanding and a general sense of the term's meaning. The knowledge scale comprises 10 items shown with the relative true/false state. Percentages of the respondents who answered correctly to each item are indicated for the participants and the non-participants groups. In grey, are the items in which participants' scores are significantly higher than non-participants, and the corresponding percentages are marked with the same white-to-green gradient used for the Understanding of the "Environmental Microbiome Sampling" process scale.

Understanding and Knowledge of the Environmental Microbioma Sampling Process - Submissions Summary

When you hear or read the term 'environmental microbiome sampling' you have

	Element 1	Element 2	Element 3	Element 4	Element 5	Element 6	Element 7	Element 8	Mean Total Score
	sterility	metadata	storage temperature	tools	microorganisms and environment	DNA	research question	replicates	
A clear understanding of what it means.									
participants - answer (N=20)	25%	10%	5%	65%	60%	20%	10%	5%	**2.00 (SD = 1.33)**
non-participants (N=5)	0%	0%	0%	20%	80%	40%	0%	0%	**1.40 (SD = 0.89)**
all respondents (N=25)	20%	8%	4%	56%	64%	24%	8%	4%	**1.88 (SD = 1.26)**
no answer (N=11)									
A general sense of what it means.									
participants (N=10)	0%	30%	0%	30%	100%	40%	20%	20%	**2.40 (SD = 1.17)**
non-participants (N=7)	0%	0%	0%	0%	43%	43%	0%	0%	**0.85 (SD = 0.89)**
all respondents (N=17)	0%	18%	0%	18%	76%	41%	12%	12%	**1.76 (SD = 1.30)**
no answer (N=15)									
Little understanding of what it means.									
participants (N=2)		Respondents with little understanding of the term were not asked to write the definition. Considered score = 0.							
non-participants (N=1)									
all respondents (N=3)									
Total participants (N=32)	16%	16%	3%	50%	69%	25%	13%	9%	**2.06 (SD = 1.31)**
Total non-participants (N=13)	0%	0%	0%	8%	54%	38%	0%	0%	**0.92 (SD = 0.91)**
Total respondents (N= 45)	11%	11%	2%	38%	64%	29%	9%	7%	**1.71 (SD = 1.30)**

Indicate whether the following affirmations are "True" or "False":

	Item 1	Item 2	Item 3	Item 4	Item 5	Item 6	Item 7	Item 8	Item 9	Item 10	Mean Total Score
	Using sterile swabs is not efficient for environmental microbiome sampling.	*Microbiome sampling includes metadata compilation.*	*Collected samples have to be stored at room temperature.*	*Collecting more replicates for each sampling point is a good practice.*	*Record the sampled surface type is not relevant because environmental microbial communities are not affected by the surface type.*	*Microbial biodiversity is higher in urban contexts than in rural contexts.*	*Without symbiotic bacteria we could not live.*	*Microorganisms are ubiquitous.*	*Microorganisms influence our health only negatively.*	*Studying environmental microbiome samples through bacterial growth on plates is the most informative approach.*	
	FALSE	TRUE	FALSE	TRUE	FALSE	FALSE	TRUE	TRUE	FALSE	FALSE	
participants (N=45)	96%	98%	98%	100%	89%	78%	96%	78%	100%	69%	**9.00 (SD = 0.95)**
non-participants (N=26)	96%	77%	62%	96%	92%	73%	96%	65%	100%	35%	**7.90 (SD = 1.12)**
all respondents (N=71)	96%	90%	85%	99%	90%	76%	96%	73%	100%	56%	**8.60 (SD = 1.13)**

Table S5. Summary of the submissions to the MATOSS and NEP subscales. Items and their scoring are shown for the two subscales and for the participants and non-participants groups. Mean scores range from -8 (anti attitudes) to 8 (pro attitudes). *Scoring for MATOSS Item 3 and Item 4 has been reversed (Strongly Agree = -2, Agree = -1, Neutral = 0, Disagree = 1, Strongly Disagree = 2).

		Attitudes toward Science *These statements refer to how you feel about science in general*					***Attitudes toward Science*** *These statements refer to how you feel about science in general*				
		**PARTICIPANTS**					**NON PARTICIPANTS**				
		Strongly Agree **2**	Agree **1**	Neutral **0**	Disagree **-1**	Strongly Disagree **-2**	Strongly Agree **2**	Agree **1**	Neutral **0**	Disagree **-1**	Strongly Disagree **-2**
**Item 1**	*Science and technology are making our lives healthier, easier, and more comfortable*	-	-	11%	53%	36%	4%	-	15%	54%	27%
**Item 2**	*The benefits of science are greater than any harmful effects*	-	16%	13%	51%	20%	4%	12%	50%	31%	4%
**Item 3***	*Science makes our way of life move too fast*	4%	18%	42%	27%	9%	8%	15%	42%	19%	15%
**Item 4***	*We depend too much of science and not enough on faith*	-	2%	18%	22%	58%	12%	-	4%	27%	58%
		**Mean Scale Score = 3.5 (standard deviation = 2.23)**					**Mean Scale Score = 0.64 (standard deviation = 0.63)**				

		Attitudes toward the Environment *These statements refer to how you feel about the relationship between humans and nature*					***Attitudes toward the Environment*** *These statements refer to how you feel about the relationship between humans and nature*				
		**PARTICIPANTS**					**NON PARTICIPANTS**				
		Strongly Agree **2**	Agree **1**	Neutral **0**	Disagree **-1**	Strongly Disagree **-2**	Strongly Agree **2**	Agree **1**	Neutral **0**	Disagree **-1**	Strongly Disagree **-2**
**Item 1**	*Humans were created to rule over the rest of nature*	80%	18%	2%	-	-	65%	23%	8%	4%	-
**Item 2**	*People have the right to modify the natural environment to suit their needs*	38%	42%	16%	4%	-	35%	38%	19%	4%	4%
**Item 3**	*Plants and animals exist primarily to be used by people*	71%	22%	4%	2%	-	73%	23%	4%	-	-
**Item 4**	*People need not to adapt to the natural environment because they can remake it to suit their needs*	60%	29%	7%	4%	-	35%	62%	4%	-	-
		**Mean Scale Score = -5.98 (standard deviation = 1.94)**					**Mean Scale Score = -5.46 (standard deviation = 2.27)**				

Additional File References

1. T. D. Paustian, et al., Development, Validation, and Application of the Microbiology Concept Inventory. Journal of Microbiology & Biology Education 18, 10.1128/jmbe.v18i3.1320 (2017).
2. D. Brossard, B. Lewenstein, R. Bonney, Scientific knowledge and attitude change: The impact of a citizen science project. International Journal of Science Education 27, 1099–1121 (2005).
3. R. E. Dunlap, Measuring Endorsement of the New Ecological Paradigm: A Revised NEP Scale.
4. N. Porticella, T. Phillips, R. Bonney, Self-Efficacy for Learning and Doing Science Scale (SELDS, Custom). Technical Brief Series. Cornell Lab of Ornithology, Ithaca NY. (2017).
